# ZhiXiaoSanZheng formula ameliorates podocyte injury in diabetic kidney disease by inhibiting ferroptosis: integrated network pharmacology and experimental validation

**DOI:** 10.1186/s13020-026-01389-x

**Published:** 2026-04-07

**Authors:** Shaofeng Zhou, Huijuan Zheng, Xinghua Zhang, Chenhui Xia, Weimin Jiang, Yaotan Li, Jiale Zhang, Shiwei Ruan, Binhua Ye, Weiwei Sun, Yaoxian Wang

**Affiliations:** 1 Department of Nephrology and Endocrinology, Dongzhimen Hospital, Beijing University of Chinese Medicine, Beijing, 100700 China; 2 Key Laboratory of Chinese Internal Medicine of Ministry of Education and Beijing, Beijing, 100700 China; 3 Department of Endocrinology, The People’s Hospital Affiliated to Fujian University of Traditional Chinese Medicine, Fuzhou, China; 4 Renal Research Institution of Beijing University of Chinese Medicine, Beijing, 100700 China

**Keywords:** ZhiXiaoSanZheng formula, Diabetic kidney disease, Podocyte, Ferroptosis, NRF2, Network pharmacology

## Abstract

**Background:**

Diabetic kidney disease (DKD) is widely recognized as a major contributor to end-stage renal disease, in which podocyte injury serves as an important pathological basis for disease progression. ZhiXiaoSanZheng Formula (ZXSZF), an empirically derived traditional Chinese medicine prescription, has shown therapeutic potential in DKD; however, its molecular mechanisms remain unclear. This study investigated whether ZXSZF protects podocytes by modulating ferroptosis-related pathways.

**Methods:**

The chemical profile of ZXSZF was analyzed by LC–MS/MS. Potential bioactive compounds were screened through SwissADME, and putative targets were predicted using SwissTargetPrediction. Overlapping targets among ZXSZF, DKD, and ferroptosis were identified and analyzed through protein–protein interaction and functional enrichment analyses. The predicted mechanisms were further validated in a unilateral nephrectomy plus STZ-induced DKD rat model and in AGEs–stimulated MPC5 podocytes.

**Results:**

LC–MS/MS analysis identified 94 chemical constituents in ZXSZF. Network pharmacology analysis suggested that antioxidant and ferroptosis-related pathways centered on NRF2 may represent potential regulatory nodes of ZXSZF. In DKD rats, ZXSZF reduced albuminuria and improved renal histopathological changes, accompanied by restoration of podocyte markers and attenuation of ferroptosis-associated alterations. In AGEs-stimulated podocytes, ZXSZF decreased lipid peroxidation and iron accumulation while enhancing cellular antioxidant capacity. These effects were associated with increased NRF2 signaling and upregulation of SLC7A11 and GPX4. Pharmacological inhibition of NRF2 with ML385 partially attenuated the protective effects of ZXSZF.

**Conclusions:**

ZXSZF alleviates podocyte injury in DKD and its renoprotective effects are associated with modulation of ferroptosis-related processes involving the NRF2/SLC7A11/GPX4 pathway. The present study provides experimental evidence for the mechanistic basis of ZXSZF and supports its potential role as a complementary therapeutic option in DKD management.

**Graphical abstract:**

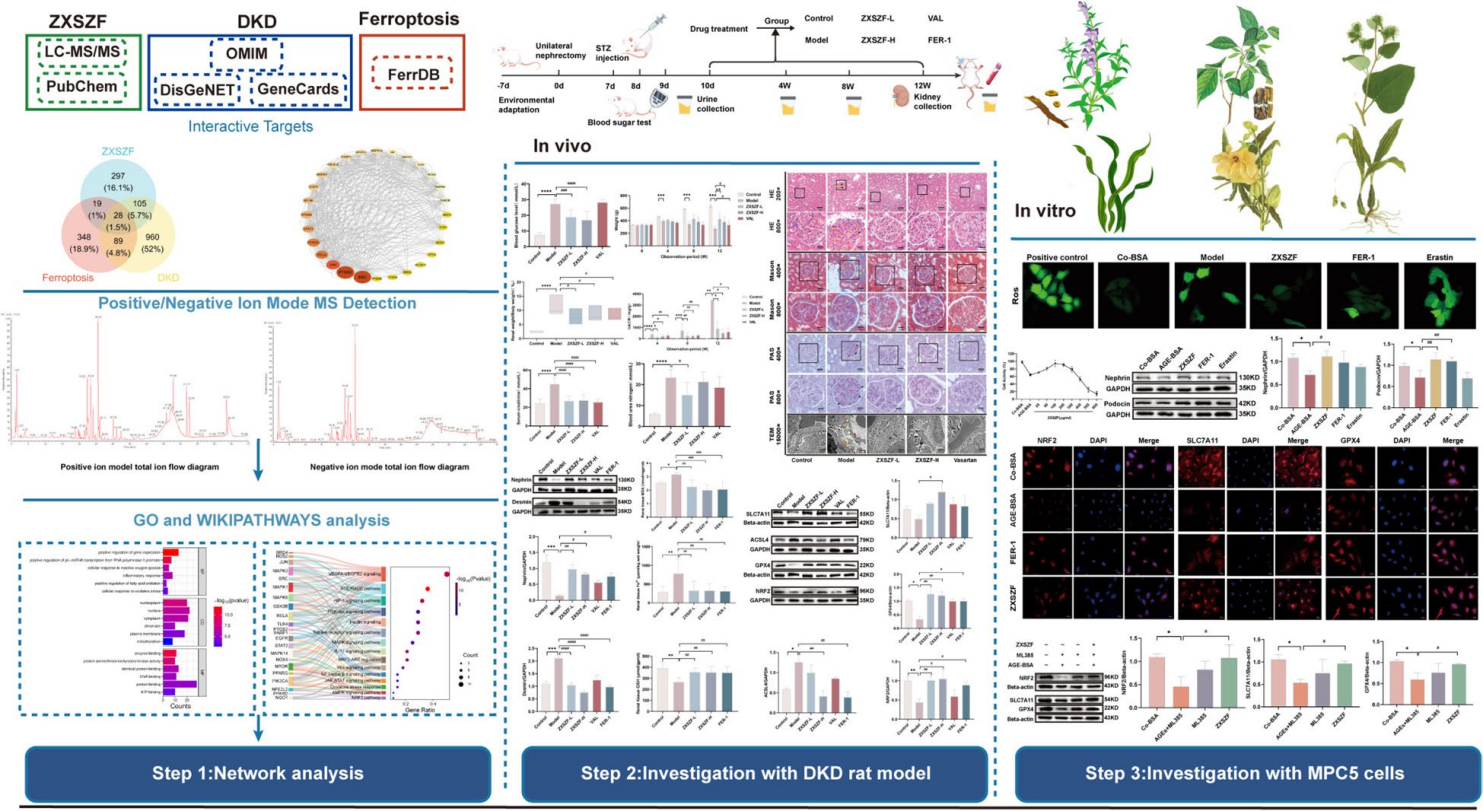

**Supplementary Information:**

The online version contains supplementary material available at 10.1186/s13020-026-01389-x.

## Introduction

Diabetic kidney disease (DKD) is a major chronic complication of diabetes characterized by microvascular injury and remains a principal contributor to end-stage renal failure globally. With the increasing global burden of diabetes, DKD develops in approximately one-third of affected individuals during disease progression [1, 2]. Current management primarily relies on controlling blood glucose and blood pressure, combined with agents targeting the renin–angiotensin system as well as newer therapies such as sodium–glucose cotransporter-2 inhibitors and glucagon-like peptide-1 receptor agonists [3, 4]. Although these interventions can delay disease progression, they rarely reverse established renal damage[5]. Many patients still develop persistent proteinuria and progressive loss of renal function. Therefore, identifying new therapeutic targets and effective treatment strategies for DKD remains an urgent clinical need.

Podocytes are terminally differentiated epithelial cells responsible for maintaining the structural and functional stability of the glomerular filtration barrier. Their interdigitating foot processes and slit diaphragm structure maintain the integrity of glomerular filtration. In the diabetic milieu, podocyte injury is promoted by multiple pathogenic factors, including metabolic imbalance, oxidative damage, and increased accumulation of advanced glycation end products (AGEs) [5–7]. These changes lead to foot process effacement, detachment, and decreased expression of structural proteins such as nephrin and podocin. As a result, the filtration barrier is disrupted and proteinuria develops. Accumulating studies suggest that podocyte damage plays a pivotal role during the early stage of DKD development and contributes substantially to disease progression [8]. Therefore, strategies aimed at protecting podocytes have become an important focus in DKD research.

Emerging evidence has implicated ferroptosis as a contributing mechanism in podocyte damage under DKD conditions [9]. This process is characterized as an iron-dependent mode of regulated cell death associated with excessive lipid peroxidation. In diabetic kidneys, persistent metabolic abnormalities drive excessive production of reactive oxygen species (ROS) via mitochondrial pathways, overwhelming the antioxidant defense system and causing oxidative damage to cell membranes and proteins, ultimately triggering podocyte ferroptosis [10]. A central regulatory mechanism of ferroptosis involves the system xc − /GSH/GPX4 axis. This transporter complex, consisting of SLC7A11 and SLC3A2, imports cystine to support intracellular glutathione generation [11]. GPX4 then reduces lipid peroxides and protects cells from ferroptosis. Nuclear factor erythroid 2–related factor 2 (NRF2) functions as a key transcriptional regulator involved in cellular antioxidant defense and iron homeostasis. Activation of NRF2 can upregulate SLC7A11 and GPX4 expression and suppress ferroptosis [12]. However, the specific regulatory mechanisms by which NRF2 modulates ferroptosis in podocytes under diabetic conditions remain incompletely elucidated. Thus, exploring the ferroptosis mechanisms of podocytes in diabetes and identifying therapeutic agents that target these pathways may provide new strategies to improve diabetic kidney injury.

Traditional Chinese medicine (TCM) has gained increasing attention in DKD management owing to its integrative pharmacological features involving multiple bioactive constituents and therapeutic targets. Herbal formulas may regulate multiple pathological processes, including oxidative stress, inflammation, and metabolic imbalance. ZhiXiaoSanZheng formula (ZXSZF) is a traditional prescription developed based on the “Shen-Luo-Zheng-Jia” theory [13]. With a long history of use in DKD management, ZXSZF has shown promising therapeutic effects. The herbal constituents of ZXSZF are shown in Table 1. Botanical classification was verified using World Flora Online (www.worldfloraonline.org), and all medicinal materials were identified according to the Pharmacopoeia of the People’s Republic of China (https://ydz.chp.org.cn) to ensure standardized source documentation. Previous clinical investigations have shown that ZXSZF, guided by the therapeutic principles of clearing heat, resolving dampness, dissipating masses, and replenishing qi, contributes to improved renal function and reduced proteinuria in patients with DKD [14]. As the sovereign herb in ZXSZF, *Astragalus membranaceus (Fisch.)* Bunge and its representative active constituents, including calycosin and astragaloside IV, have been reported to exert protective effects in DKD. Experimental evidence further indicates that these constituents reduce 24-h urinary total protein (24 h-UTP) and serum creatinine levels, accompanied by reduced oxidative stress and ferroptosis-related damage [15, 16]. Another key component of ZXSZF, *Abelmoschus manihot (L.) Medik.* and its ethanolic extract such as *Huangkui capsules* reduce DKD-related proteinuria by preserving mitochondrial function and mitigating podocyte injury [17, 18]. In addition, our previous study found that *Chinese Pholidota Herb* (*Pholidota chinensis*) significantly improved hyperglycemia, proteinuria, and lipid metabolism disorders in DKD, and also exerted protective effects on renal function. These findings have been documented in our patented work on the application of Chinese Pholidota Herb in the preparation of drugs for DKD treatment (CN117338875B). Together, these results suggest that ZXSZF may exert renal protective effects through multiple pharmacological pathways. However, the molecular mechanisms underlying its action, particularly its potential role in regulating ferroptosis during DKD progression, remain unclear.

**Table 1:** The information on herbal medicines in ZXSZF

Number	Chinese name	English name	Botanical Plant Names	Part used	Voucher number	Dose (g)
1	Shi Xian Tao	Pholidota Herb	*Pholidota chinensis Lindl*	Dried whole plant or pseudobulb	Quanzhou 9,920,241,107,001	50
2	Huang Qi	Astragalus Root	*Astragalus mongholicus Bunge*	Dried Root	Shandong7170104	30
3	Huang Shu Kui Hua	Abelmoschus Flower	*Abelmoschus manihot (L.) Medik*	Dried Flower	Hunan701060901	30
4	Niu Bang Zi	Great Burdock Achene	*Arctium lappa L*	Dried Fruit	Hebei701020202	9
5	Du Zhong	Eucommia Bark	*Eucommia ulmoides Oliv*	Dried Bark	Sichuan7170206	10
6	Hai Zao	Seaweed	*Sargassum pallidum (Turn.) C. Ag*	Dried algae	Zhejiang7130121	30
7	Shui Zhi	Leech	*Hirudo nipponica Whitman*	Dried animal body	Jiangshu7121102	6

The botanical names of herbs in No. 1–5 have been checked via World Flora Online (https://www.worldfloraonline.org/); those in No. 6–7 have been checked with “Chinese Pharmacopoeia” (https://ydz.chp.org.cn)

In recent years, systems biology-based strategies have become important tools for elucidating the pharmacological basis of complex herbal prescriptions. Network analysis can help identify potential bioactive compounds and candidate targets associated with disease pathways [19]. However, computational predictions require experimental validation. Here, liquid chromatography-tandem mass spectrometry (LC–MS/MS) together with network-based analysis was employed to characterize the candidate metabolites of ZXSZF and explore their potential associations with DKD and ferroptosis-related targets. Experimental validation was then carried out in both in vivo and in vitro models to clarify whether ZXSZF protects podocytes by modulating ferroptosis through the NRF2/SLC7A11/GPX4 signaling pathway.

## Materials and methods

### Network analysis

**Fig. 1:**
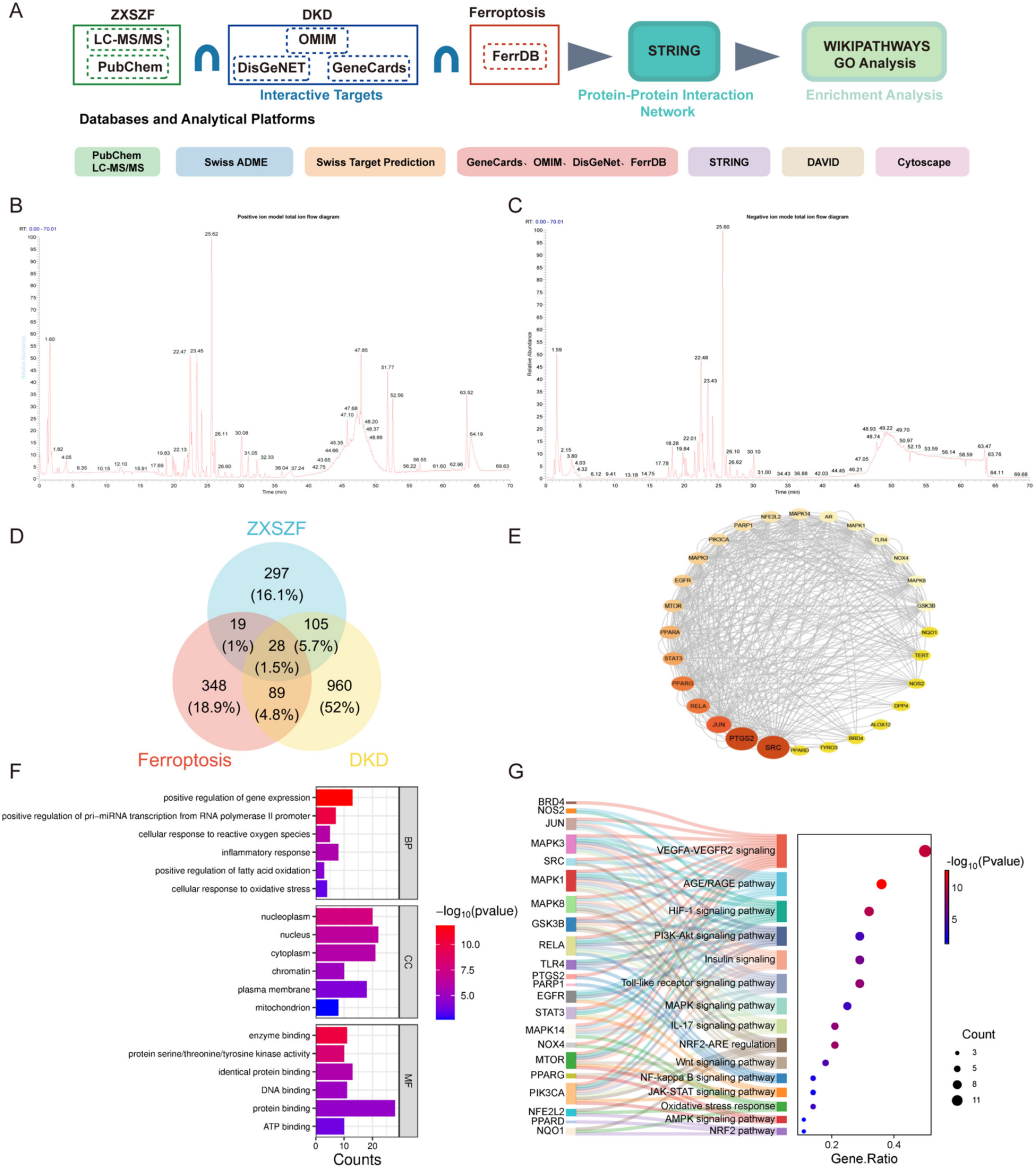
Network analysis and pathway prediction of ZXSZF in DKD-related ferroptosis. **A** Workflow of compound identification, target prediction, overlap analysis, PPI construction, and enrichment analysis. **B**, **C** Total ion chromatograms of ZXSZF detected by LC–MS/MS in positive and negative ion modes. **D** Venn diagram showing overlapping targets among ZXSZF, DKD, and ferroptosis. **E** PPI network of 28 shared targets. **F** GO enrichment analysis of overlapping targets. **G** WIKIPATHWAYS enrichment analysis of enriched signaling pathways. Abbreviations: BP, biological process; CC, cellular component; MF, molecular function; PPI, protein–protein interaction

A flowchart of network analysis is shown in Fig. 1A. First, the 94 compounds identified by LC–MS/MS were filtered using the SwissADME platform: only those with high gastrointestinal absorption and a bioavailability score ≥ 0.5 were retained, resulting in 48 metabolites. Next, the structures of these metabolites were uploaded to the SwissTargetPrediction database for target prediction, and 449 targets with a probability > 0.5 were selected. For disease and mechanism-related targets, DKD-related targets were collected from the GeneCards (https://www.genecards.org/), OMIM (https://omim.org/), and DisGeNet databases (https://www.disgenet.org/), with 1182 unique targets obtained after deduplication; ferroptosis-related targets were retrieved from the FerrDB database (http://www.zhounan.org/ferrdb/), totaling 484. Through Venn diagram analysis, 28 overlapping targets were identified, which were defined as “potential associated targets” without any assumption of “activity”. These 28 shared targets were then imported into the STRING database for protein–protein interaction (PPI) analysis, generating a network composed of 28 nodes and 402 edges. Finally, functional enrichment analysis was performed using the DAVID database, with thresholds set as *P* < 0.05 and FDR < 0.05 for Gene Ontology (GO) analysis, and *P* < 0.01 for WIKIPATHWAYS analysis.

### Chemicals and reagents

Creatinine (C011-2-1), urinary microalbumin (C035-2-1), blood urea nitrogen (C013-2-1), glutathione (A006-2-1), and malondialdehyde (A003-1-1) were obtained from Nanjing Jiancheng Bioengineering Institute (Nanjing, China). Iron concentration detection kits (E-BC-K773-M), Cell Ferrous Iron Colorimetric Assay Kit (E-BC-K881-M-48 T), and ROS Fluorescence Assay Kit (E-BC-K138-F) were purchased from Elabscience (Wuhan, China). RPMI 1640 medium (11,875,093/11879020, 1:1 dilution) was sourced from Gibco (New York, USA), fetal bovine serum (WS500T) from Austin (Shanghai, China), and penicillin/streptomycin (P1400) from Solarbio (Beijing, China). AGE-bovine serum albumin (AGE-BSA, 22,968-10) was provided by Cayman (Michigan, USA). Cell Counting Kit-8 (CCK-8, GK10001) and ferroptosis regulators (Fer-1, 1 μM, GC10380; erastin, 3 μM, GC16630) were procured from GLPBIO (Montclair, USA), while lactate dehydrogenase (LDH) solution (G1780) was from Promega (Wisconsin, USA). Phalloidin-FITC (P5282) was purchased from Sigma-Aldrich (St. Louis, Missouri, USA). The NRF2 inhibitor ML385 (5 μM, M00240) was obtained from BioLab (Beijing, China). An overview of the primary antibodies used in this study is presented in Supplementary Table 1.

### Preparation and composition analysis of ZXSZF

ZXSZF was sourced from Dongzhimen Hospital at the Beijing University of Chinese Medicine and the quality of these herbal materials was verified by the hospital’s TCM pharmacy. Botanical drugs in ZXSZF were taxonomically validated using the Plants of the World Online database (http://www.plantsoftheworldonline.org/) and the Medicinal Plant Names Services database (MPNS, http://mpns.kew.org/mpns-portal/). Detailed information on the botanical drugs included in the formula is presented in Table 1. For preparation, the crude herbal materials were first soaked in distilled water at a drug-to-solvent ratio of 1:10 (w/v) for 1 h at room temperature. The mixture was then decocted twice, with each decoction performed for 1 h. The two filtrates were pooled and concentrated until the extract reached an equivalent crude drug concentration of 1 g/mL. The extraction yield was calculated as 15.6% using the formula: dry extract weight divided by the initial crude drug weight multiplied by 100%. The rat dosage was determined based on body surface area conversion principles for extrapolating human equivalent doses, with reference to previously published methods [20]. Briefly, the low dose of ZXSZF was 6.3 × 165 g/70 kg based on a human-rat equivalent dose ratio of 6.3, with a clinical dosage of 165 g/day for a 70 kg adult. The high dose group received twice the amount of the low-dose group. For cell experiments, the aqueous extract of ZXSZF was further processed by lyophilization to obtain a stable powder. Freeze-drying was conducted at − 80 °C under a vacuum pressure of 0.1 mbar. The resulting powder was stored in airtight containers at − 20 °C until use. LC–MS/MS was employed to profile the major chemical constituents present in the ZXSZF extract. The detailed parameters and analytical procedures for mass spectrometry are described in Supplementary Method 1.

### Animals

Male Sprague–Dawley rats aged 5–7 weeks were obtained from Beijing Weitong Lihua Laboratory Animal Technology Co., Ltd. (Beijing, China; Certificate No.SCXK(HU)2016-0011). Animals were allowed to adapt for one week prior to intervention in a controlled environment maintained at 24 ± 2 °C and 60% ± 5% humidity under alternating 12 h light and dark periods, with food and water available ad libitum. Beijing University of Chinese Medicine approved the study protocol (BUCM-2022111002-4084).

### DKD model and experimental design

To induce DKD, rats were subjected to unilateral nephrectomy combined with streptozotocin (STZ) exposure. After left nephrectomy, animals received an intraperitoneal injection of STZ (55 mg/kg) freshly prepared in 0.1 mol/L sodium citrate buffer (pH 4.5). For the sham group, rats were given an equivalent volume of citrate buffer intraperitoneally without nephrectomy. Fasting glucose concentrations were determined three days after STZ injection, and animals with levels above 16.7 mmol/L were defined as diabetic and enrolled in subsequent study [21, 22]. The animals were randomly divided into six groups (n = 10 per group): control group, model group, ZXSZF low-dose group (ZXSZF-L, 14.85 g/kg), ZXSZF high-dose group (ZXSZF-H, 29.7 g/kg), valsartan group (VAL, 8 mg/kg), and a ferroptosis inhibitor group treated with Ferrostatin-1 (Fer-1, 2.5 mg/kg). ZXSZF extract and valsartan were dissolved in distilled water and administered once daily by oral gavage for 12 weeks. Fer-1 was administered intraperitoneally according to previously reported protocols for ferroptosis inhibition [23]. Animals in the control and model groups were administered the same volume of saline.

**Fig. 2:**
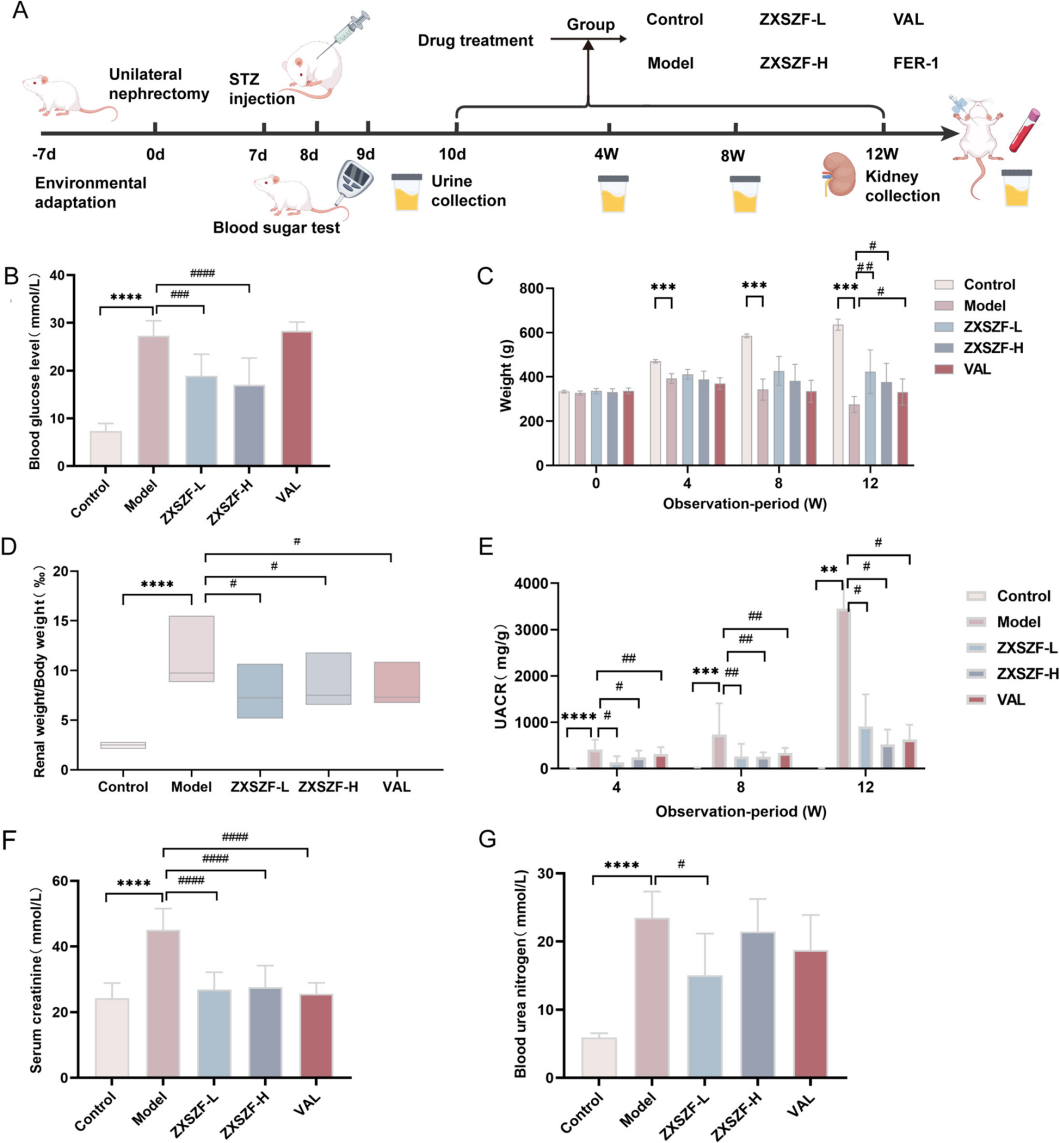
ZXSZF ameliorates metabolic abnormalities and renal dysfunction in DKD rats. **A** Schematic diagram of the experimental protocol for DKD induction and drug intervention. **B** Blood glucose levels after 12 weeks of treatment. **C** Body weight changes during the intervention period. **D** Kidney index at week 12. **E** Urinary albumin-to-creatinine ratio (UACR) at weeks 4, 8, and 12. **F**, **G** Serum creatinine and blood urea nitrogen levels at the end of treatment. Data are expressed as mean ± SD, except for panel D presented as median with interquartile range [M (P25, P75)]. Statistical analysis was performed using one-way ANOVA for B and E–G, repeated-measures ANOVA for C, and Kruskal–Wallis test for D. n = 6. **P < 0.01, ***P < 0.001, ****P < 0.0001 versus control group; #*P* < 0.05, ##*P* < 0.01, ###*P* < 0.001, ####*P* < 0.0001 versus model group. Notes: ZXSZF-L, ZXSZF low-dose group; ZXSZF-H, ZXSZF high-dose group; VAL, valsartan group

Body weight and blood glucose were recorded once per week throughout the experiment (Fig. 2A). At the end of the experimental period, urine samples were collected from fasted rats. The animals were then euthanized, and blood samples were collected from the abdominal aorta for biochemical analysis. Kidney tissues were excised and weighed, and the renal index was calculated as kidney weight (g) divided by body weight (g) × 100%.

### Biochemical analysis

Urinary creatinine levels were determined using an enzymatic method, while urinary microalbumin was measured by immunoturbidimetry. The urinary albumin-to-creatinine ratio (UACR) was subsequently calculated. Serum creatinine, blood urea nitrogen (BUN), and blood glucose in rats were quantified using commercially available reagents following the corresponding protocols. Biochemical analyses were completed using an automated analyzer (AU5800, Beckman Coulter, USA) at the Department of Laboratory Medicine, Dongzhimen Hospital.

### Histological examination

Kidney samples were first fixed in 4% paraformaldehyde, followed by ethanol gradient dehydration, paraffin embedding, and sectioning into 4 μm slices. The prepared sections were stained with hematoxylin and eosin (H&E), periodic acid–Schiff (PAS), and Masson's trichrome to evaluate renal histopathology. Morphological alterations were assessed microscopically, and representative fields were recorded for further analysis. For transmission electron microscopy (TEM), small kidney specimens (approximately 1 mm^3^) were fixed in 2.5% glutaraldehyde followed by osmium tetroxide fixation and processed for ultrastructural examination.

### Measurement of MDA, Fe^2^⁺, and GSH

Frozen renal tissues (100 mg) were homogenized and the supernatants were collected for analysis. Ferrous iron (Fe^2^⁺) content was measured with a commercial iron detection kit. Malondialdehyde (MDA) and total glutathione (T-GSH) levels were measured in kidney homogenates using commercial assay kits according to the manufacturers’ protocols.

### Western blot analysis

Proteins from kidney tissues or cultured podocytes were extracted with RIPA buffer containing protease and phosphatase inhibitors. After centrifugation, the supernatant was collected and protein concentration was determined with a BCA assay kit. Equal amounts of denatured protein were separated by SDS-PAGE, transferred onto PVDF membranes, and blocked with 5% BSA. The membranes were then incubated with primary antibodies at 4 °C overnight, followed by TBST washing and incubation with HRP-linked secondary antibodies for 1 h at room temperature. Protein signals were detected by enhanced chemiluminescence (ECL), and band intensity was quantified using ImageJ software.

### Cell culture

Conditionally immortalized mouse podocytes (MPC5) were maintained in RPMI-1640 medium containing 10% FBS, 100 U/mL penicillin/streptomycin, and 10 U/mL recombinant mouse IFN-γ. Cells were maintained at 33 °C in a humidified incubator containing 5% CO_2_. To induce differentiation, podocytes were transferred to non-permissive conditions (37 °C) without IFN-γ and cultured for 10–14 days.

### Cell viability and cytotoxicity assays

Based on previous research from our group, differentiated MPC5 cells (4000 cells/well) were exposed to AGE-BSA (200 μg/mL) for 24 h to mimic podocyte injury in DKD. An equal concentration of nonglycated Co-BSA was used as the control [24].

For viability assessment, each well received 10 μL of CCK-8 solution followed by incubation at 37 °C for 2 h. Absorbance was measured at 450 nm. For cytotoxicity detection, 10 μL of 10 × lysis buffer was added to positive control wells 45 min before measurement. Then, 50 μL of each sample was moved to a fresh plate and combined with an equal volume of CytoTox 96® reagent. Following 30 min incubation at room temperature in the dark, stop solution (50 μL) was added before recording absorbance at 490 nm.

### Phalloidin staining

After PBS washing, cells were fixed in 4% paraformaldehyde, permeabilized with 0.2% Triton X-100, and blocked using 5% goat serum. FITC-conjugated phalloidin (2 μg/mL, Cytoskeleton) was then applied for 1 h at room temperature in the dark, followed by DAPI nuclear staining. Fluorescence images were obtained with a TCS SP5 II confocal microscope (Leica).

### Detection of ROS, MDA, and Fe^2+^

Podocytes (5 × 10^5^ cells/mL) were seeded in 6-well plates. Intracellular ROS was evaluated by DCFH-DA staining (1:5000) after incubation at 37 °C for 10 min, followed by serum-free medium washing and fluorescence observation. Signal intensity was quantified using ImageJ software. Intracellular MDA and Fe^2^⁺ levels were measured with commercial assay kits, with absorbance for MDA recorded at 532 nm using a spectrophotometer (Thermo Fisher, Finland).

### Immunofluorescence

Differentiated MPC5 cells (6000 cells/well) were plated in 24-well plates and exposed to the indicated treatments for 24 h after cell attachment. Following PBS washing, cells were fixed with 4% paraformaldehyde, permeabilized using 0.2% Triton X-100, and blocked with 5% goat serum. Primary antibodies were applied overnight at 4 °C, followed by incubation with matched fluorescent secondary antibodies for 1 h in the dark. After DAPI staining, fluorescence images were collected under a fluorescence microscope.

### Statistical analysis

All assays were independently repeated at least three times. Statistical analysis and graphical outputs were completed using GraphPad Prism 9.3.0. Data with normal distribution are presented as mean ± SEM, whereas non-normally distributed variables are summarized as median with interquartile range [M (P25, P75)]. Distribution characteristics were evaluated using the Shapiro–Wilk test before group comparisons. For two-group comparisons, an independent-samples t-test was applied. Multiple-group comparisons were conducted using one-way ANOVA followed by LSD post hoc testing when parametric assumptions were satisfied, whereas variables without normal distribution, including kidney index, were analyzed using the Kruskal–Wallis test. A two-sided *P* value < 0.05 was considered statistically significant.

## Results

### Network analysis

A total of 94 compounds were identified by LC–MS/MS analysis. All identified chemical constituents are presented in Table 2, and corresponding total ion chromatogram (TIC) is shown in Fig. 1B, C. After screening via the SwissADME platform, 48 metabolites were retained, including hyperoside, arctigenin, isoquercitrin, and calycosin. Intersection analysis between DKD-related targets and ferroptosis-associated genes identified 28 overlapping targets (Fig. 1D). A PPI network generated from the 28 shared targets consisted of 28 nodes linked by 402 edges (Fig. 1E). Key nodes included NFE2L2 (NRF2), JUN, and STAT3. GO enrichment results suggested that these targets were primarily associated with biological processes related to oxidative stress response and lipid metabolic regulation (Fig. 1F). WIKIPATHWAYS enrichment analysis further indicated significant enrichment in the NRF2 signaling pathway (Fig. 1G). These results provided preliminary clues for subsequent experimental validation.

**Table 2:** Identification of chemical constituents in the extract of ZXSZF by UHPLC-QE Orbitrap MS/MS

NO	RT (min)	Molecular formula	Calc.MW	EV(m/z)	Error (ppm)	Secondary fragment (MS/MS)	Ms2Adduct	Compound type	Compound name	References
1	22.642	C₂₁ H₂₀ O₁₂	464.09576	462.08	0.62	302.03857, 301.03464, 300.02774, 283.02493, 271.02499	[M-H]^−^	Flavonol glycoside	Hyperoside	[45–47]
2	25.796	C₂₁ H₂₄ O₆	372.15741	373.1648	0.32	323.12845, 306.12164, 305.11725, 295.13284, 291.10071	[M + H]^+^	Lignan	Arctigenin	[43, 44]
3	22.615	C₁₅ H₁₀ O₇	302.04286	303.0501	0.7	285.03952, 274.04776, 257.04456, 247.05975, 230.05302	[M + H]^+^	Flavonol	Morin	[45–47]
4	1.791	C₁₂ H₂₂ O₁₁	342.11621	341.1087	− 0.01	180.05943, 179.05627, 161.04547, 149.04550, 143.03497	[M + CI]^−^	Disaccharide	Sucrose	[48, 49]
5	1.81	C₇ H₇ N O₂	137.0479	136.062	1.61	136.06204, 129.07431, 119.03552, 110.06032	[M + H]^+^	Alkaloid	Trigonelline HCl	[52–54]
6	24.341	C₂₁ H₂₀ O₁₂	464.09581	463.0883	0.71	303.04117, 302.03882, 300.02829, 273.04028, 257.04654	[M-H]^−^	Flavonol glycoside	Isoquercitrin	[45–47]
7	3.97	C₆ H₈ O₇	192.027	191.0197	− 0.02	173.00896, 154.99843, 147.12270, 147.02946	[M-H]^−^	Organic acid	Citric acid	[48, 49]
8	1.747	C₅ H₁₁ N O₂	117.07912	118.0864	1.21	118.08638, 102.05515, 91.05440, 74.06036	[M + H]^+^	Quaternary ammonium alkaloid	Betaine	[50, 51]
9	22.205	C₂₇ H₃₀ O₁₆	610.15347	609.1467	0.14	445.08295, 343.04626, 302.03717, 301.03372, 299.02106	[M-H]^−^	Flavonol glycoside	Rutin	[45–47]
10	1.805	C₅ H₉ N O₂	115.06353	116.0708	1.78	116.07079, 115.13168, 114.12791, 98.09647, 98.06006	[M + H]^+^	Amino acid	2-Pyrrolidinecarboxylic acid	[50, 51]
11	18.484	C₁₆ H₂₂ O₁₀	374.12126	373.114	− 0.1	211.06104, 209.03090, 193.05006, 167.07132	[M-H]^−^	Iridoid glycoside	Geniposidic acid	[55, 56]
12	2.012	C₅ H₁₁ N O₂	117.07913	118.0864	1.26	118.08638, 116.07085, 102.05502, 91.05438	[M + H]^+^	Amino acid	L-Valine	[50, 51]
13	12.318	C₁₀ H₁₃ N₅ O₄	267.0971	268.1043	1.31	242.92711, 137.06638, 137.04626, 119.03548	[M + H]^+^	Nucleoside	Adenosine	[50, 51]
14	20.119	C₁₆ H₁₈ O₉	354.09508	353.0879	0	192.05936, 191.05620, 179.03506, 174.04919	[M-H]^−^	Organic acid	Cryptochlorogenic acid	[43, 44]
15	24.59	C₁₅ H₁₀ O₈	318.03763	317.0302	0.21	299.02032, 289.03528, 272.02902, 271.02408, 255.03008	[M-H]^−^	Flavonol	Myricetin	[45–47]
16	26.826	C₁₅ H₁₀ O₇	302.04268	301.0353	0.09	274.04459, 257.04587, 255.03107, 245.04568	[M-H]^−^	Flavonol	Quercetin	[45–47]
17	1.707	C₆ H₁₄ O₆	182.07897	181.0717	− 0.38	180.05975, 179.05603, 163.06122, 162.04898	[M + CI]^−^	Sugar alcohol	Mannitol	[48, 49]
18	5.185	C₆ H₁₃ N O₂	131.09488	130.9667	1.93	130.08684, 129.01846, 125.96148, 113.96410	[M + H]^+^	Amino acid	L-Leucine	[50, 51]
19	22.313	C₂₂ H₂₂ O₁₀	446.12157	447.1292	0.61	393.21408, 286.07941, 285.07605, 270.05249, 253.04979	[M + H]^+^	Isoflavone glycoside	Calycosin-7-O-β-D-glucoside	[52–54]
20	23.875	C₂₅ H₂₄ O₁₂	516.12701	515.1195	0.46	354.08759, 353.08813, 335.07901, 255.06535	[M-H]^−^	Organic acid	Isochlorogenic acid C	[43, 44]
21	23.11	C₂₅ H₂₄ O₁₂	516.12697	515.1196	0.38	354.09009, 353.08838, 335.07709, 317.03024	[M-H]^−^	Organic acid	Isochlorogenic acid B	[43, 44]
22	14.946	C₁₀ H₁₃ N₅ O₅	283.09176	282.0844	0.33	151.04660, 151.02567, 150.04208, 126.03065	[M-H]^−^	Nucleoside	Isoguanosine	[50, 51]
23	1.794	C₇ H₁₂ O₆	192.06331	191.0561	− 0.42	191.02066, 173.04530, 171.02966, 155.03470,	[M-H]^−^	Organic acid	Quinic acid	[43, 44]
24	25.02	C₂₂ H₂₂ O₉	430.12669	431.1346	0.71	361.02768, 360.02994, 359.02798, 271.08600, 269.08102	[M + H]^+^	Isoflavone glycoside	Ononin	[52–54]
25	20.698	C₁₇ H₂₄ O₁₀	388.13699	387.1299	0.11	225.11388, 225.07753, 208.10655	[M + FA-H]^−^	Iridoid glycoside	Geniposide	[55, 56]
26	23.493	C₂₅ H₂₄ O₁₂	258.06339	515.1198	1.78	354.08820, 353.08835, 335.07574	[2M-H]^−^	Organic acid	1,3-Dicaffeoylquinic acid	[43, 44]
27	20.904	C₃₂ H₄₂ O₁₆	682.24759	681.2402	0.45	359.14014, 358.13800, 357.13470, 342.11093, 311.12933	[M + FA-H]^−^	Lignan glycoside	Pinoresinol diglucoside	[55, 56]
28	26.806	C₁₆ H₁₂ O₅	284.0687	285.0762	0.79	283.22354, 270.05237, 269.04462, 254.05305	[M + H]^+^	Isoflavone	Calycosin	[52–54]
29	6.313	C₉ H₁₂ N₂ O₆	244.06949	243.0622	− 0.18	243.0629, 201.05913, 200.05669, 153.02995	[M-H]^−^	Nucleoside	Uridine	[50, 51]
30	24.311	C₉ H₁₆ O₄	188.10477	187.0975	− 0.47	186.11339, 170.09053, 169.08705, 159.87883,	[M-H]^−^	Aliphatic dicarboxylic acid	Azelaic acid	[48, 49]
31	21.432	C₂₈ H₃₂ O₁₆	624.17003	625.1772	1.6	480.11932, 479.11761, 318.06915, 317.06580	[M + H]^+^	Flavonol glycoside	Narcissoside	[45–47]
32	31.108	C₁₆ H₁₂ O₄	268.0738	269.0812	0.89	254.05707, 253.04970, 237.05484, 226.06256	[M + H]^+^	Isoflavone	Formononetin	[52–54]
33	1.832	C₄ H₄ O₄	116.01078	115.0035	− 1.55	115.00362, 114.93378, 85.02941, 74.02446	[M-H]^−^	Organic acid	Maleic acid	[48, 49]
34	1.812	C₆ H₆ O₃	126.03193	127.0392	1.83	126.05520, 109.02858	[M + H]^+^	Furan derivative	5-Hydroxymethylfurfural	[52–54]
35	23.492	C₂₅ H₂₄ O₁₂	516.1277	517.1353	1.8	319.08578, 315.05484, 186.05519	[M + H-H20]^+^	Organic acid	3,5-Dicaffeoylquinic acid	[43, 44]
36	3.164	C₆ H₅ N O₂	123.03226	124.0396	1.92	124.03954, 123.05547, 122.06844, 112.03947	[M + H]^+^	Alkaloid	Nicotinic acid	[50, 51]
37	5.994	C₉ H₈ O₃	164.04763	165.0549	1.72	163.03928, 147.04420, 137.09622, 137.05992	[M + NH4]^+^	Phenolic acid	p-Coumaric acid	[55, 56]
38	3.301	C₄ H₅ N₃ O	111.04343	113.9639	1.5	108.95840, 104.96352	[M + H]^+^	Nitrogenous base	Cytosine	[50, 51]
39	18.482	C₁₀ H₈ O₃	176.0477	177.055	2.02	159.04430, 150.06355, 147.96941, 149.06000	[M + H]^+^	Coumarin	7-Methoxycoumarin	[40–42]
40	20.819	C₉ H₈ O₄	180.04228	179.0349	0.11	178.02312, 164.01158, 149.02475, 137.05040, 136.04848	[M-H]^−^	Phenolic acid	Caffeic acid	[43, 44]
41	41.956	C₁₈ H₃₀ O₂	278.22493	279.2322	1.26	261.22067, 223.16959, 209.15451,	[M + H]^+^	Fatty acid	α-Linolenic acid	[48, 49]
42	23.631	C₁₆ H₁₂ O₇	316.05849	319.0443	0.58	302.04236, 290.04282, 274.04630, 263.05573	[M + H]^+^	Flavonol	Isorhamnetin	[45–47]
43	19.554	C₇ H₆ O₃	138.03166	137.0243	− 0.27	136.86276, 136.01660, 119.01394, 110.03289	[M-H]^−^	Phenolic aldehyde	Protocatechualdehyde	[55, 56]
44	23.203	C₂₆ H₃₂ O₁₁	520.19462	519.1873	0.3	491.18887, 359.14047, 358.13788, 357.13470, 342.11050	[M-H]^−^	Lignan glycoside	Pinoresinol 4-O-glucoside	[55, 56]
45	24.974	C₇ H₆ O₃	138.03163	137.0243	− 0.47	138.01945, 136.86279, 108.02158	[M-H]^−^	Phenolic acid	4-Hydroxybenzoic acid	[55, 56]
46	24.779	C₂₄ H₂₄ O₁₁	488.13309	489.1403	2.52	416.21475, 286.07941, 285.07605, 270.05246, 253.04988	[M + H]^+^	Isoflavone glycoside	6''-O-Acetylglycitin	[52–54]
47	26.231	C₂₃ H₂₈ O₁₀	464.16858	463.1613	0.71	302.11160, 301.10812, 301.03571, 286.08487, 271.06125	[M-H]^−^	Isoflavan glycoside	Isomucronulatol 7-O-glucoside	[52–54]
48	2.862	C₁₈ H₃₂ O₁₆	504.16945	503.1623	0.83	263.07782, 222.07047, 221.06671	[M + FA-H]^−^	Oligosaccharide	Manninotriose	[48, 49]
49	21.141	C₃₃ H₄₄ O₁₇	712.25841	711.2514	0.79	389.15121, 388.14868, 372.12158, 358.10110, 357.09787	[M + FA-H]^−^	Lignan glycoside	( −)-Syringaresnol-4-O-β-D-apiofuranosyl-(1 → 2)-β-D-glucopyranoside	[55, 56]
50	3.158	C₅ H₅ N₅	135.05449	134.0472	− 0.06	134.04721, 132.86787, 115.00355, 108.03971, 107.03623	[M-H]^−^	Nitrogenous base	Adenine	[50, 51]
51	18.026	C₇ H₆ O₄	154.02656	153.0193	− 0.29	152.89513, 142.94717, 138.03206, 123.04523	[M-H]^−^	Phenolic acid	Protocatechuic acid	[43, 44]
52	39.584	C₁₇ H₃₄ O₂	316.26124	315.254	0.61	297.24359, 279.23346, 269.25052, 253.25500, 219.84647	[M-H]^−^	Fatty acid ester	Methyl hexadecanoate	[48, 49]
53	21.079	C₂₀ H₂₃ N O₄	341.16317	342.1705	1.36	323.12729, 298.11670, 297.11252, 267.06549, 266.09000	[M + H]^+^	Alkaloid	( +)-Magnoflorine	[40–42]
54	25.869	C₉ H₁₀ O₃	166.06316	167.0704	0.99	165.09120, 165.05504, 152.04706, 151.03932, 139.07559	[M + H]^+^	Aromatic aldehyde	o-Veratraldehyde	[55, 56]
55	20.709	C₉ H₈ O₂	148.05275	149.06	2.14	149.05994,149.02361,131.04973,121.06501,121.03983	[M + H]^+^	Phenylpropanoid acid	Cinnamic acid	[43, 44]
56	21.345	C₇ H₆ O₂	122.03662	121.0294	− 1.31	121.02940,108.02168,107.01707	[M-H]^−^	Phenolic aldehyde	p-Hydroxybenzaldehyde	[55, 56]
57	2.684	C₇ H₁₀ O₅	174.05274	173.0091	− 0.5	172.06181, 155.03503, 143.03493, 137.02440, 129.01930	[M-H]^−^	Organic acid	Shikimic acid	[40–42]
58	21.153	C₉ H₆ O₂	146.03697	147.0443	1.33	146.05991, 128.04987, 121.02859, 119.08562	[M + H]^+^	Coumarin	Coumarin	[40–42]
59	19.249	C₁₆ H₁₇ N O₃	254.09446	272.1285	− 1.31	256.10474, 240.07826, 226.06175, 209.09619	[M + NH4]^+^	Alkaloid	Higenamine	[40–42]
60	20.119	C₁₅ H₁₄ O₆	290.07905	289.0717	0.05	247.06142, 247.02553, 245.08194, 227.07207, 221.08205	[M-H]^−^	Flavanol	Epicatechin	[45–47]
61	23.495	C₂₁ H₂₀ O₁₁	448.1008	447.0936	0.53	329.13889, 327.05099, 286.04269, 285.03918, 284.03284	[M-H]^−^	Flavonol glycoside	Astragalin	[45–47]
62	30.208	C₄₁ H₆₈ O₁₄	830.46669	829.4595	0.79	119.03483, 113.02445	[M-H]^−^	Triterpenoid saponin	Astragaloside IV	[52–54]
63	19.739	C₂₆ H₂₈ O₁₅	580.14372	581.1512	1.56	580.22565, 289.06033, 288.05853	[M + H]^+^	Flavanol glycoside	Leucoside	[45–47]
64	19.841	C₂₁ H₂₁ Cl O₁₁	448.10118	449.1083	0.79	362.02588, 361.02454, 289.06049, 288.05847, 287.05502	[M + H]^+^	Anthocyanin glycoside	Cyanidin-3-O-glucoside chloride	[45–47]
65	21.904	C₂₈ H₃₂ O₁₅	608.17528	609.1828	1.91	591, 464.12585, 463.12500, 447.08981, 429.08185	[M + H]^+^	Flavonoid glycoside	Diosmin	[45–47]
66	23.802	C₂₂ H₂₂ O₁₁	462.11683	463.1241	1.34	462.18051, 303.04977, 302.07431, 287.05109, 286.04721	[M + H]^+^	Flavone glycoside	Diosmetin-7-O-β-D-glucopyranoside	[45–47]
67	18.486	C₈ H₈ O₄	168.04211	167.0348	− 0.88	166.83331, 153.01498, 151.03993, 149.06068	[M + FA-H]^−^	Phenolic acid	4-Methoxysalicylic acid	[40–42]
68	20.302	C₁₀ H₁₀ O₄	194.05828	194.118	1.91	193.15892, 193.04955, 177.05486, 176.07060	[M + H-H20]^+^	Phenolic acid	Ferulic acid	[55, 56]
69	22.434	C₈ H₈ O₃	152.04762	153.1276	1.84	153.05492, 145.98988, 135.11705, 134.06023	[M + H]^+^	Aromatic aldehyde	2-Hydroxy-4-methoxybenzaldehyde	[40–42]
70	23.233	C₁₅ H₁₀ O₆	304.05862	287.0554	− 0.88	270.18106, 258.05295, 241.04976, 231.06531	[M + H-H20]^+^	Flavonol	Kaempferol	[45–47]
71	21.356	C₃₄ H₄₆ O₁₈	759.29595	760.3032	1.33	401.15973, 331.11600, 330.10983, 315.08591	[M + H]^+^	Lignan glycoside	( −)-Syringaresinol di-O-glucoside	[55, 56]
72	20.951	C₁₅ H₁₄ O₆	290.07912	289.0718	0.29	247.06123, 246.08467, 245.08209, 221.08214	[M-H]^−^	Flavanol	( +)-Catechin hydrate	[45–47]
73	22.16	C₈ H₈ O₃	152.04713	151.04	− 1.39	151.00360, 149.02420, 137.01965, 136.01651	[M-H]^−^	Phenolic aldehyde	Vanillin	[55, 56]
74	23.213	C₂₀ H₂₂ O₆	358.14168	357.1343	0.11	343.11249, 342.11108, 311.12912, 256.07535, 241.05078	[M-H]^−^	Lignan	( +)-Pinoresinol	[55, 56]
75	18.494	C₁₅ H₁₄ O₇	306.07418	305.0666	0.73	305.01, 261.07526, 243.06737, 221.04561, 219.06507	[M-H]^−^	Flavanol	Epigallocatechin	[45–47]
76	21.439	C₁₅ H₁₂ O₈	320.05319	319.0459	− 0.09	301.03400, 257.04581, 238.07281, 233.04515	[M-H]^−^	Flavanol	Dihydromyricetin	[45–47]
77	22.751	C₂₇ H₃₀ O₁₅	594.159	593.1516	0.89	387.14490, 327.05090, 286.04321, 285.03955	[M-H]^−^	Flavonol glycoside	Kaempferol 3-glucorhamnoside	[45–47]
78	22.397	C₂₁ H₂₀ O₁₀	432.10603	433.1136	0.9	415.10309, 397.09259, 379.08252, 368.08267, 367.08182	[M + H]^+^	Flavone C-glycoside	Isovitexin	[45–47]
79	32.938	C₂₀ H₁₈ O₈	386.10063	387.1079	1.19	372.08353, 357.06082, 341.06650, 314.04236, 311.05588	[M + H]^+^	Isoflavone	Irisflorentin	[52–54]
80	19.551	C₁₅ H₁₄ O₇	306.07395	305.0779	− 0.01	261.08926, 237.07753, 221.04524	[M-H]^−^	Flavanol	( −)-Gallocatechin	[45–47]
81	34.223	C₁₈ H₁₆ O₇	344.08998	345.0973	1.1	330.07324, 316.05362, 315.05005, 312.06302	[M + H]^+^	Flavonoid	Lysionotin	[40–42]
82	23.613	C₂₇ H₃₄ O₁₂	567.23216	568.2397	0.11	372.15244, 371.14978, 353.13898, 319.04507, 321.11169	[M + H]^+^	Lignan glycoside	Tracheloside	[43, 44]
83	23.012	C₂₇ H₃₀ O₁₅	534.13774	593.1517	0.89	433.15213, 328.05417, 327.04956, 286.04407, 285.04016	[M-H + HAc]^−^	Flavonol glycoside	Kaempferol-3-O-rutinoside	[45–47]
84	20.365	C₁₈ H₂₂ O₁₁	414.11653	459.1147	0.76	267.09668, 191.03546, 163.03970, 148.04863, 147.04514	[M + FA-H]^−^	Iridoid glycoside	Asperuloside	[55, 56]
85	2.731	C₂₄ H₄₂ O₂₁	666.22273	665.2153	1.31	425.12915, 384.12106, 341.10855, 263.07642	[M + FA-H]^−^	Oligosaccharide	Stachyose	[52–54]
86	21.578	C₁₁ H₁₀ O₃	190.06333	191.0706	1.75	189.09163, 176.04723, 164.07993, 161.05995	[M + H]^+^	Coumarin	7-Methoxy-4-methylcoumarin	[40–42]
87	20.751	C₉ H₆ O₄	178.02656	177.0193	− 0.25	162.03165, 161.02480, 149.02426, 141.86798	[M-H]^−^	Coumarin	Esculetin	[40–42]
88	23.027	C₁₁ H₁₀ O₄	174.03211	207.0656	0.89	193.04613, 179.07065, 176.04297	[M + H + MeOH]^+^	Coumarin	Citropten	[40–42]
89	19.438	C₁₈ H₂₄ O₁₂	432.12675	431.156	− 0.05	299.11447, 251.05644, 243.02979, 207.06644	[M-H]^−^	Iridoid glycoside	Asperulosidic acid	[55, 56]
90	3.636	C₁₈ H₃₂ O₁₆	550.17512	549.1679	1.31	503.15607, 221.06700, 179.05605	[M-H]^−^	Oligosaccharide	Raffinose	[52–54]
91	34.57	C₂₀ H₁₈ O₄	322.12075	323.1281	0.76	299.06174, 268.06888, 267.06540, 255.06544	[M + H]^+^	Isoflavone	Neobavaisoflavone	[52–54]
92	29.635	C₁₆ H₁₂ O₆	300.06367	299.0562	0.92	285.03616, 256.03732, 255.02933, 239.03539	[M-H]^−^	Flavone	Hydroxygenkwanin	[45–47]
93	21.958	C₃₂ H₃₈ O₁₉	726.20167	725.1944	1.3	286.04144, 285.03882, 255.02995, 227.03502	[M-H]^−^	Flavonoid glycoside	Camelliaside B	[45–47]
94	36.27	C_45_ H_72_ O_16_	868.48204	913.4808	0.6	834.56751, 815.65565, 801.64272, 702.51105	[M + FA-H]^−^	Triterpenoid saponin	Astragaloside I	[52–54]

### ZXSZF improved hyperglycemia, body weight loss, and kidney index in DKD rats

The experimental design is shown in Fig. 2A. After STZ injection, DKD rats developed persistent hyperglycemia throughout the study. After 12 weeks of treatment, blood glucose levels were reduced to different extents in the ZXSZF-L, ZXSZF-H, and valsartan groups compared with the model group (Fig. 2B). Over the 12-week observation period, body weight increased gradually in the control group but declined in the model group. Relative to the model group, rats treated with ZXSZF-L or ZXSZF-H showed improved body weight at weeks 8 and 12, whereas the valsartan group showed a less stable trend (Fig. 2C). The kidney index was significantly higher in the model group than in the control group (*P* < 0.0001). This increase was significantly attenuated by ZXSZF-L, ZXSZF-H, and valsartan treatment (all *P* < 0.05; Fig. 2D).

### ZXSZF improved renal function in DKD rats

Renal function was evaluated based on UACR, serum creatinine, and BUN levels. Compared with the control group, UACR was significantly increased in the model group at week 4 and continued to rise at weeks 8 and 12. In contrast, ZXSZF-L, ZXSZF-H, and valsartan significantly reduced UACR levels during the intervention period (Fig. 2E). Relative to the control group, the model group showed obvious increases in serum creatinine and BUN levels. Treatment with ZXSZF or valsartan significantly decreased both indices, with no significant difference between the ZXSZF-treated groups and the valsartan group (Fig. 2F, G).

### ZXSZF improved pathological changes in kidney tissues of DKD rats

**Fig. 3:**
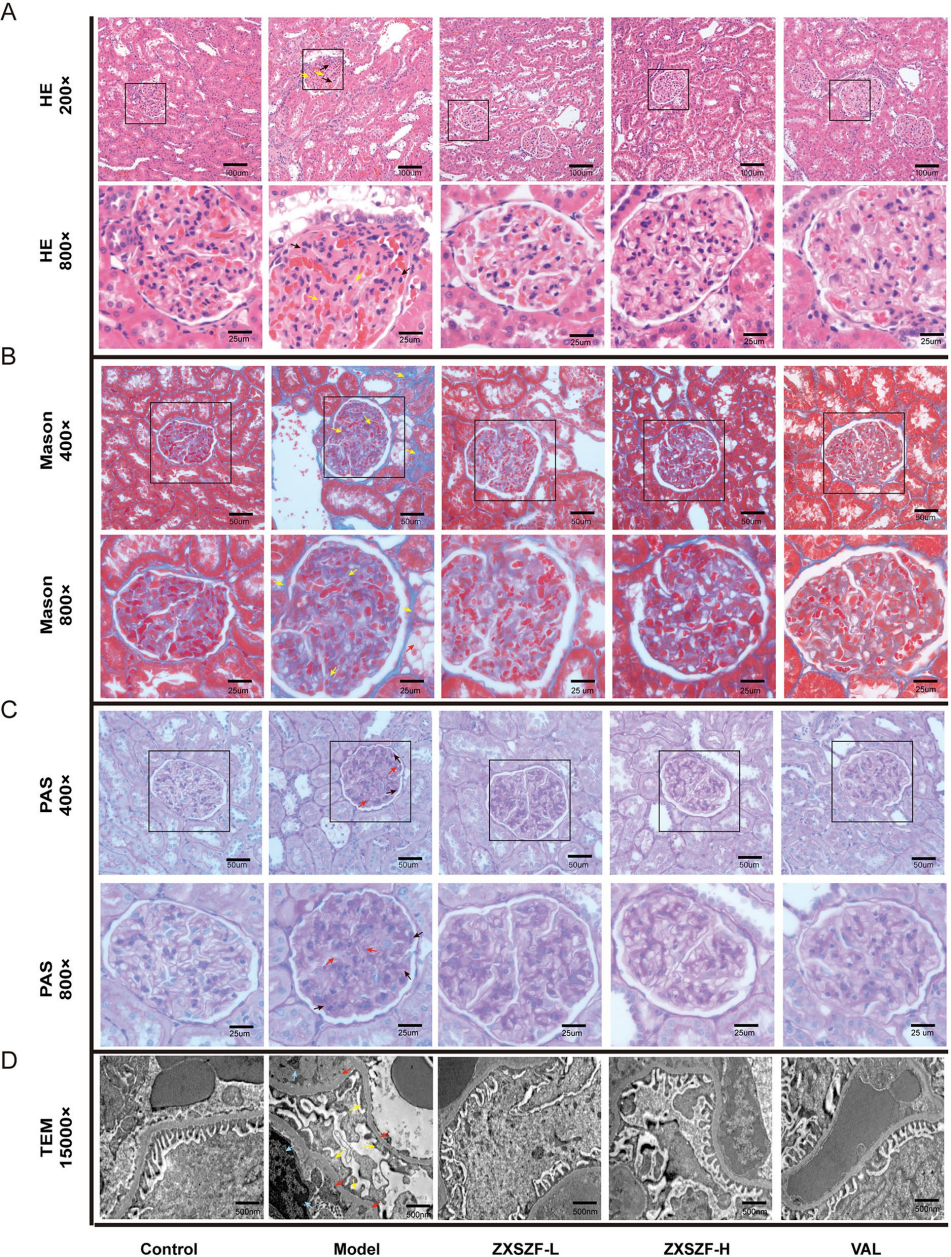
ZXSZF improves renal histopathological injury in DKD rats. **A** Representative hematoxylin and eosin (H&E) staining of kidney tissues at 200 × and 800 × magnification. **B** Representative Masson’s trichrome staining showing collagen deposition at 400 × and 800 × magnification. **C** Representative periodic acid–Schiff (PAS) staining of glomerular structure at 400 × and 800 × magnification. **D** Representative transmission electron microscopy images of glomerular ultrastructure (15,000 × magnification). Scale bars: 100 μm and 25 μm in A; 50 μm and 25 μm in B and C; 500 nm in D. Notes: ZXSZF-L, ZXSZF low-dose group; ZXSZF-H, ZXSZF high-dose group; VAL, valsartan group

Histological examination showed that glomeruli in the control group had normal structure without apparent atrophy or sclerosis. In contrast, the model group exhibited enlarged glomeruli accompanied by atrophy and sclerosis. Treatment with ZXSZF-L, ZXSZF-H, or valsartan alleviated these pathological alterations (Fig. 3A). Masson's trichrome staining revealed little collagen deposition in control kidneys, whereas obvious collagen accumulation and fibrotic alterations were observed in the model group. These changes were reduced after treatment with ZXSZF-L, ZXSZF-H, or valsartan (Fig. 3B). PAS staining showed intact glomerular architecture without mesangial expansion in the control group. In the model group, glomerulosclerotic features were observed, including mesangial matrix expansion, extracellular matrix deposition, and the presence of Kimmelstiel-Wilson nodules. Treatment with ZXSZF-L, ZXSZF-H, or valsartan reduced these structural alterations and mesangial proliferation (Fig. 3C). TEM further revealed characteristic ultrastructural injury in the model group, manifested by glomerular basement membrane thickening, podocyte foot process effacement, and reduced podocyte number. These ultrastructural abnormalities were alleviated after treatment with ZXSZF-L, ZXSZF-H, or valsartan (Fig. 3D).

### ZXSZF protected against podocyte injury in DKD rats

**Fig. 4:**
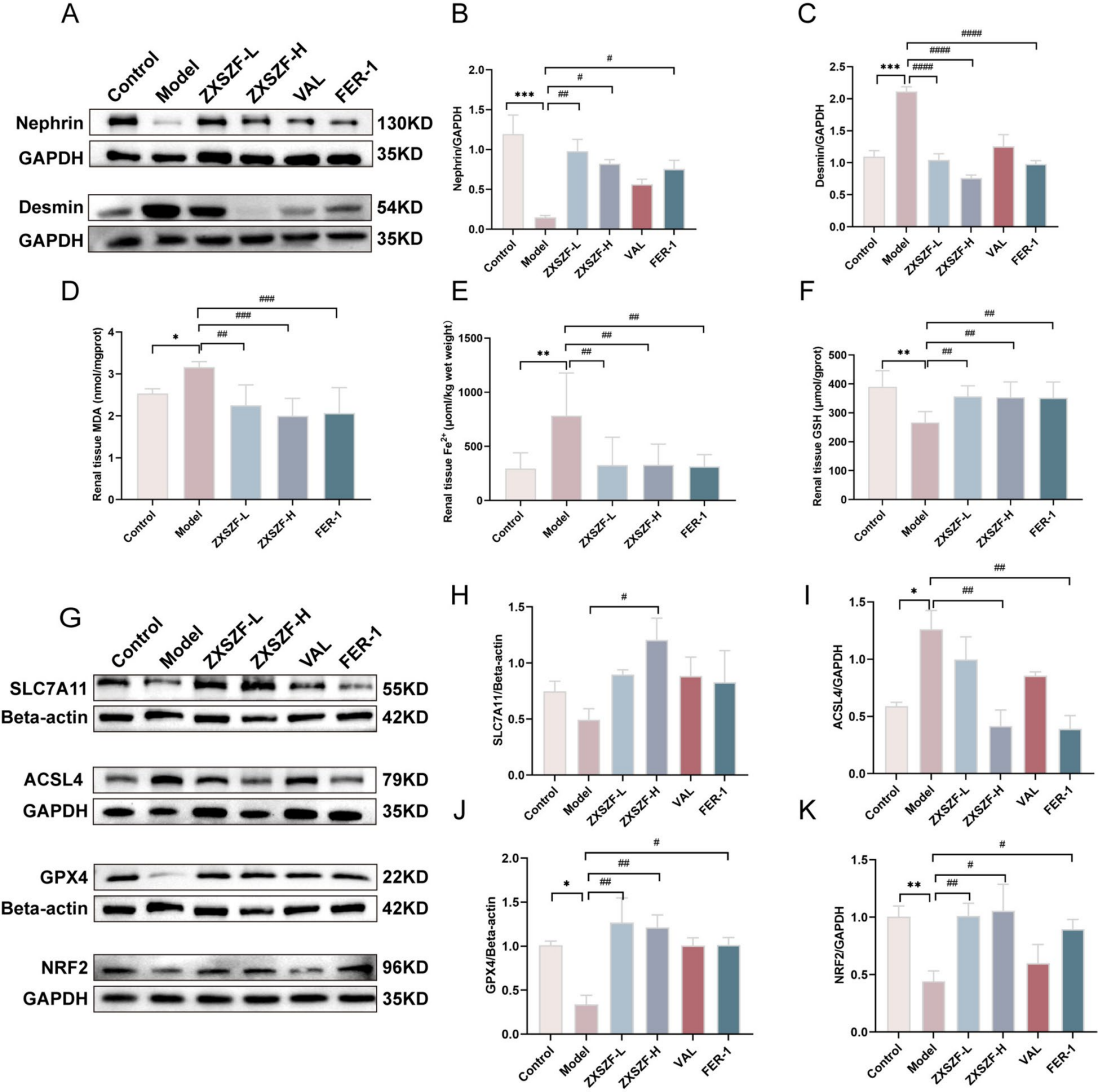
ZXSZF attenuates podocyte injury and ferroptosis in DKD rats. **A** Western blot analysis of nephrin and desmin expression in renal tissues. **B**, **C** Quantitative analysis of nephrin and desmin protein levels. **D–F** Renal malondialdehyde (MDA), ferrous iron (Fe^2^⁺), and glutathione (GSH) levels. **G** Western blot analysis of SLC7A11, ACSL4, GPX4, and NRF2 expression in renal tissues. **H–K** Quantitative analysis of SLC7A11, ACSL4, GPX4, and NRF2 protein levels. Data are expressed as mean ± SD. Statistical analysis was performed by one-way ANOVA. n = 3 for B, C and H–K; n = 6 for D–F. *P < 0.05, **P < 0.01, ***P < 0.001 versus control group; #*P* < 0.05, ##*P* < 0.01, ###*P* < 0.001, ####*P* < 0.0001 versus model group. Notes: ZXSZF-L, ZXSZF low-dose group; ZXSZF-H, ZXSZF high-dose group; VAL, valsartan group; Fer-1, ferrostatin-1 group

Downregulation of nephrin disrupts the glomerular filtration barrier, whereas increased desmin expression reflects podocyte injury[25]. In comparison with controls, the model group exhibited reduced nephrin expression and increased desmin levels (both *P* < 0.05). Treatment with ZXSZF-L, ZXSZF-H, or valsartan partially reversed these changes (all *P* < 0.05). Fer-1, used as a ferroptosis inhibitor control, showed a similar pattern with increased nephrin and reduced desmin expression (Fig. 4A–C).

### ZXSZF reduced renal lipid peroxidation and iron accumulation in DKD rats

As a hallmark of ferroptosis, lipid oxidative stress is commonly accompanied by disturbed redox balance, with GSH and MDA serving as representative indicators [11]. Relative to controls, renal tissues from DKD rats exhibited elevated MDA (*P* < 0.05) and Fe^2^⁺ (*P* < 0.01) levels together with reduced GSH content (*P* < 0.01). Administration of ZXSZF-L, ZXSZF-H, or Fer-1 reversed these alterations, as reflected by lower MDA and Fe^2^⁺ levels (both *P* < 0.01) and increased GSH levels (*P* < 0.01). No obvious differences were detected among the intervention groups (Fig. 4D–F).

### ZXSZF modulated ferroptosis-related proteins in DKD rats

GPX4 and SLC7A11 are components of the system Xc⁻/GSH/GPX4 axis, which regulates ferroptosis [26]. As an important transcriptional regulator, NRF2 governs antioxidant responses together with genes related to ferroptosis [12]. Compared with controls, NRF2 (*P* < 0.01) and GPX4 (*P* < 0.05) expression were significantly decreased in the model group, whereas SLC7A11 displayed a decreasing tendency without reaching statistical significance (*P* > 0.05). In contrast, ACSL4 expression was elevated (*P* < 0.05). Treatment with ZXSZF-L, ZXSZF-H, or Fer-1 increased NRF2, SLC7A11, and GPX4 expression while reducing ACSL4 levels compared with the model group (Fig. 4G–K). These results indicate that the renoprotective action of ZXSZF in DKD is closely associated with modulation of ferroptosis-related proteins.

### ZXSZF improved viability and cytoskeletal structure of AGEs-stimulated MPC5 cells

**Fig. 5:**
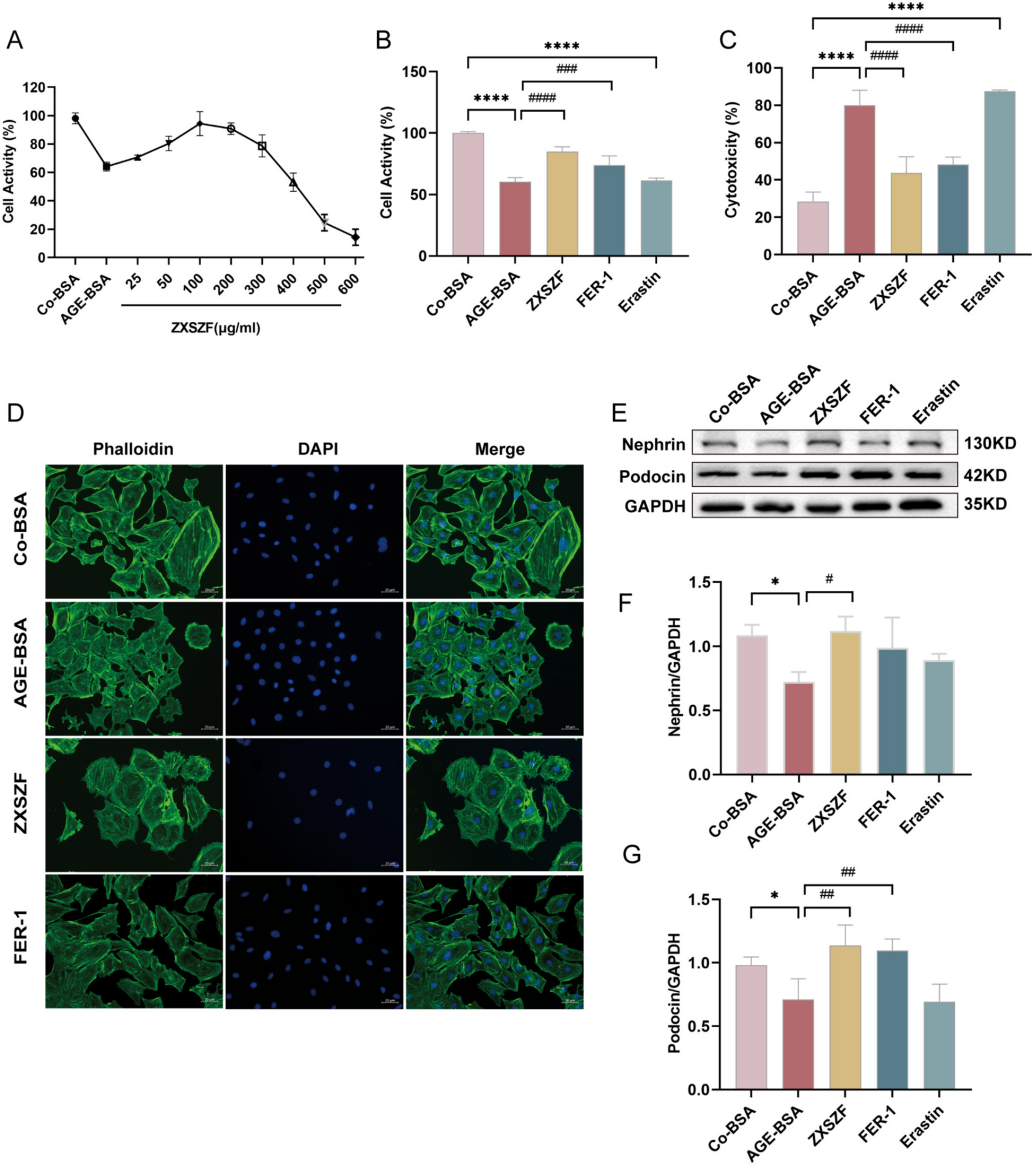
ZXSZF ameliorates AGEs-induced podocyte injury in MPC5 cells. **A** Concentration screening of ZXSZF based on cell viability in MPC5 cells exposed to AGEs. **B**, **C** Cell viability and cytotoxicity assessed by CCK-8 and LDH assays. **D** Representative phalloidin staining of F-actin cytoskeleton in MPC5 cells. **E** Western blot analysis of nephrin and podocin expression. **F**, **G** Quantitative analysis of nephrin and podocin protein levels. Data are expressed as mean ± SD. Statistical analysis was performed by one-way ANOVA. n = 3. *P < 0.05, ****P < 0.0001 versus Co-BSA group; #*P* < 0.05, ##*P* < 0.01, ###*P* < 0.001, ####*P* < 0.0001 versus AGE-BSA group. Notes: Co-BSA, control bovine serum albumin group; AGE-BSA, advanced glycation end product-modified bovine serum albumin group; Fer-1, ferrostatin-1 group; Erastin, ferroptosis inducer group

MPC5 cells were treated with AGEs for 24 h to generate an in vitro model of DKD-associated podocyte injury, according to our previous protocol [24]. Compared with the control group, AGEs exposure reduced podocyte viability (*P* < 0.01). Concentration screening showed that ZXSZF at 50–200 μg/mL improved the decrease in cell viability induced by AGEs, with 100 μg/mL showing the most consistent effect. Therefore, 100 μg/mL ZXSZF was selected for subsequent experiments (Fig. 5A).

Further evaluation of cell viability and cytotoxicity showed that AGEs treatment reduced podocyte viability and increased LDH release compared with the control group, showing a pattern similar to that observed with the ferroptosis inducer erastin. By contrast, ZXSZF treatment improved cell viability while lowering LDH release compared with the model group. A similar pattern was observed in the ferroptosis inhibitor Fer-1 group (Fig. 5B, C). Phalloidin staining showed that AGEs exposure disrupted actin cytoskeletal organization and altered cell morphology in MPC5 cells. These structural changes were alleviated after treatment with ZXSZF or Fer-1 (Fig. 5D).

Nephrin and podocin are key proteins involved in maintaining slit diaphragm structure and filtration barrier stability [25]. Western blot results demonstrated that AGE-BSA exposure led to lower nephrin and podocin expression than that observed in the control group (both *P* < 0.05). ZXSZF treatment increased nephrin (*P* < 0.05) and podocin (*P* < 0.01) expression relative to the model group. In contrast, protein levels in the erastin group were similar to those in the model group, whereas Fer-1 treatment increased nephrin and podocin expression (Fig. 5E–G).

### ZXSZF reduced AGEs-induced ferroptosis in MPC5 cells

**Fig. 6:**
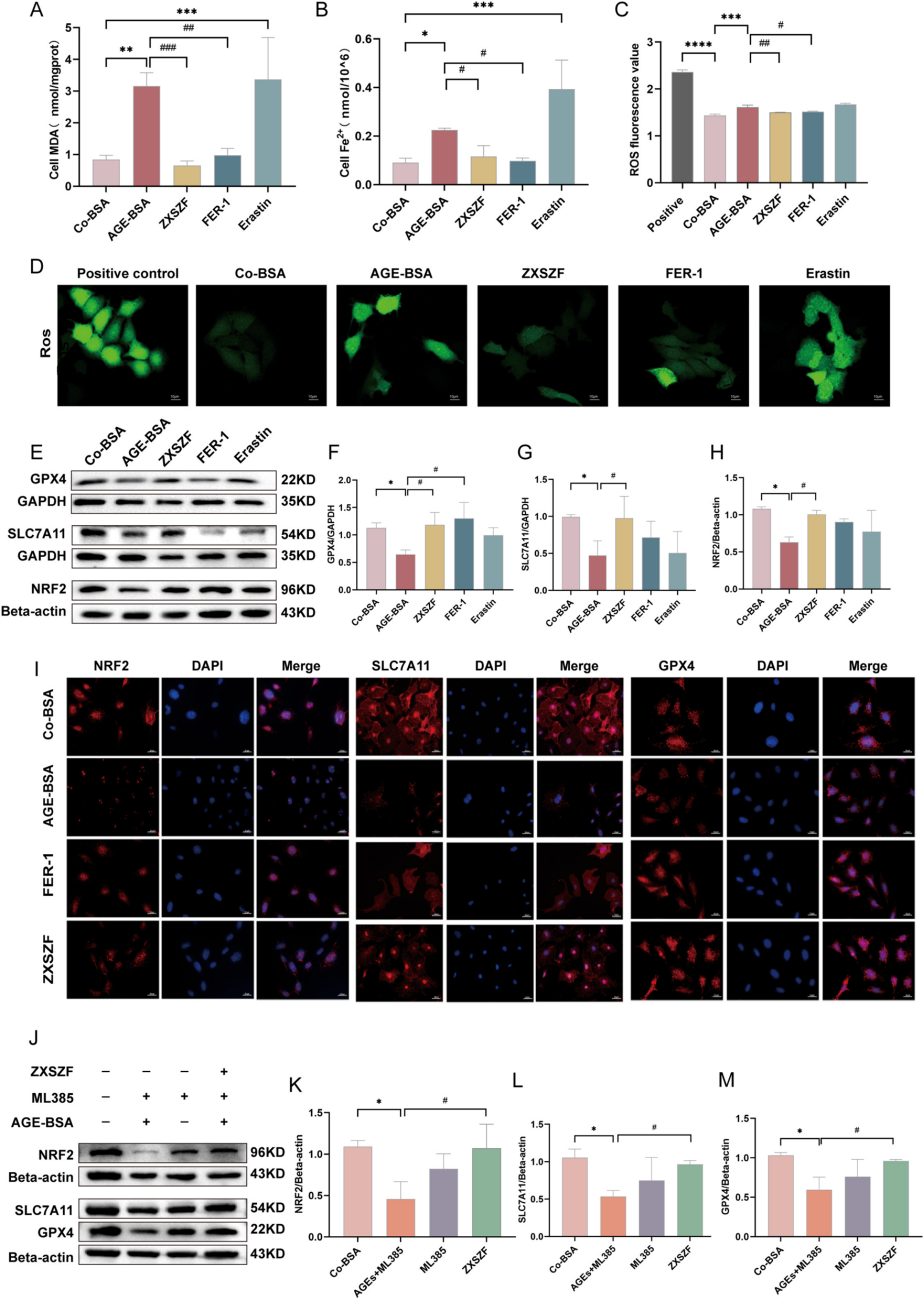
ZXSZF attenuates ferroptosis through the NRF2/SLC7A11/GPX4 pathway in AGEs-stimulated MPC5 cells. **A**, **B** Cellular malondialdehyde (MDA) and ferrous iron (Fe^2^⁺) levels. **C** Quantitative analysis of intracellular ROS levels. **D** Representative fluorescence images of ROS staining. **E** Western blot analysis of GPX4, SLC7A11, and NRF2 expression. **F**–**H** Quantitative analysis of GPX4, SLC7A11, and NRF2 protein levels. **I** Representative immunofluorescence staining of NRF2, SLC7A11, and GPX4 in MPC5 cells. **J** Western blot analysis of NRF2, SLC7A11, and GPX4 after ML385 intervention. **K**–**M** Quantitative analysis of NRF2, SLC7A11, and GPX4 protein levels after ML385 treatment. Scale bars: 10 μm in D; 20 μm in I. Data are expressed as mean ± SD. Statistical analysis was performed by one-way ANOVA. n = 3. *P < 0.05, **P < 0.01, ***P < 0.001, ****P < 0.0001 versus Co-BSA group; #*P* < 0.05, ##*P* < 0.01, ###*P* < 0.001 versus AGE-BSA group. Notes: Co-BSA, control bovine serum albumin group; AGE-BSA, advanced glycation end product-modified bovine serum albumin group; Fer-1, ferrostatin-1 group; Erastin, ferroptosis inducer group; ML385, NRF2 inhibitor

To further examine ferroptosis-related changes in vitro, several biochemical indicators were measured in MPC5 cells. As shown in Fig. 6A, MDA levels were higher in the AGEs-induced model group (*P* < 0.01) and in the erastin group (*P* < 0.001) compared with the control group. Treatment with ZXSZF (*P* < 0.001) or Fer-1 (*P* < 0.01) reduced MDA levels relative to the model group.

Cellular ferrous ion levels showed a similar pattern (Fig. 6B). Compared with the control group, Fe^2^⁺ levels were increased in both the model group (*P* < 0.05) and the erastin group (*P* < 0.001). ZXSZF and Fer-1 treatment reduced Fe^2^⁺ accumulation compared with the model group (both *P* < 0.05).

ROS production was also evaluated (Fig. 6C, D). Fluorescence intensity and quantified values were higher in the model and erastin groups than in the control group (*P* < 0.01). Treatment with ZXSZF or Fer-1 reduced ROS levels compared with the model group (*P* < 0.05).

Western blot analysis showed that the model group had lower expression of NRF2 (*P* < 0.05), SLC7A11 (*P* < 0.05), and GPX4 (*P* < 0.05) compared with the control group, with a similar pattern observed in the erastin group (Fig. 6E–H). ZXSZF treatment increased NRF2, SLC7A11, and GPX4 expression relative to the model group (all *P* < 0.05). Fer-1 increased GPX4 expression (*P* < 0.05), while changes in SLC7A11 were less evident. Immunofluorescence staining showed a similar distribution pattern for NRF2, SLC7A11, and GPX4 (Fig. 6I).

### ZXSZF influenced ferroptosis-related proteins through NRF2 in MPC5 cells

Based on the network analysis and in vivo results, the involvement of NRF2 was further examined using the NRF2 inhibitor ML385. In the AGEs + ML385 group, NRF2 protein levels were lower than in the control group (*P* < 0.05), whereas ZXSZF treatment increased NRF2 expression relative to the AGEs + ML385 group (*P* < 0.05) (Fig. 6J, K). Consistent changes were observed for SLC7A11 and GPX4. Both proteins were reduced in the AGEs + ML385 group compared with the control group (P < 0.05). ZXSZF treatment increased SLC7A11 and GPX4 expression relative to the AGEs + ML385 group (*P* < 0.05) (Fig. 6J, L, M). Collectively, this supports that ZXSZF alleviates ferroptosis in AGEs-induced podocytes by regulating the Nrf2/SLC7A11/GPX4 pathway (Fig. 6J–M).

## Discussion

DKD represents one of the most serious microvascular complications associated with diabetes and remains a principal contributor to end-stage renal failure worldwide. In this study, we investigated the therapeutic effects of the TCM formula ZXSZF in DKD models and explored its potential mechanism in relation to ferroptosis. Our findings indicate that ZXSZF improved renal function, mitigated pathological injury, and protected podocytes in DKD rats. These effects were accompanied by decreased lipid peroxidation and iron accumulation as well as regulation of ferroptosis-related proteins. In vitro experiments further showed that ZXSZF improved the viability and structural integrity of AGEs-stimulated podocytes and was associated with modulation of NRF2-related ferroptosis signaling. Together, these findings suggest that the renoprotective effects of ZXSZF in DKD may be associated with the regulation of ferroptosis-related processes.

In the present study, ZXSZF improved several physiological and renal functional indicators in DKD rats. Treatment reduced blood glucose levels, partially restored body weight, and decreased the kidney index. Renal indicators including UACR, serum creatinine, and BUN were also improved following ZXSZF administration. Histological evaluation further demonstrated less glomerular hypertrophy, mesangial matrix expansion, and collagen accumulation in diabetic kidneys following intervention. These findings indicate that ZXSZF alleviates both functional and structural renal injury under diabetic conditions. Consistent with these observations, Chen et al. reported that TCM formula improved renal function and attenuated pathological damage in DKD rats [22]. The present findings therefore provide further experimental evidence supporting the renoprotective effects of this prescription in DKD.

Podocyte injury is a key pathological event in DKD progression because disruption of the slit diaphragm and cytoskeleton directly contributes to albuminuria and glomerular filtration barrier dysfunction [6]. In the current study, DKD rats exhibited reduced nephrin expression together with increased desmin levels, indicating structural damage to podocytes. ZXSZF treatment partially restored nephrin expression and improved cytoskeletal organization. In AGEs-stimulated MPC5 cells, ZXSZF also improved cell viability and preserved cytoskeletal structure. These findings suggest that ZXSZF contributes to maintaining podocyte structural integrity under diabetic conditions. Similar protective effects on podocytes were reported by Wu et al., who showed that the Chinese herbal compound alleviated renal injury in DKD and improved podocyte-associated markers while activating mitophagy-related pathways [27]. These observations further support the protective effects of this formulas on podocyte structure and renal filtration function.

The renoprotective effects of ZXSZF may also be associated with the pharmacological activities of its constituent herbs. Astragalus membranaceus exerts direct cytoprotective effects on podocytes through bioactive components such as astragaloside IV, which maintains podocyte structural integrity and reduces proteinuria [28, 29]. *Abelmoschus manihot (L.) Medik.*, another key component of the formula, and its extract have been reported to mitigate renal inflammation and glomerular injury [30]. In addition, arctigenin from *Fructus Arctii* attenuates proteinuria and podocyte damage in diabetic mice [31]. These individual contributions of ZXSZF’s constituent herbs are likely to act synergistically, collectively contributing to the observed podocyte protection. Such multi-component and multi-target characteristics reflect the pharmacological features of traditional Chinese medicine formulas.

Ferroptosis has been increasingly recognized as a contributor to renal injury in DKD, characterized by iron overload, lipid oxidative damage, and impaired antioxidant defense [32]. In the present study, DKD rats and AGEs-treated podocytes exhibited increased MDA and Fe^2^⁺ levels together with decreased GSH levels, indicating enhanced oxidative stress and lipid peroxidation. ZXSZF treatment reversed these biochemical alterations. At the molecular level, ZXSZF influenced the expression of NRF2, SLC7A11, and GPX4, which are critical regulators of ferroptosis. These findings are consistent with previous reports indicating that dysregulation of SLC7A11, GPX4, and ACSL4 contributes to ferroptosis-associated renal injury in DKD [21].

It should be noted that the involvement of the NRF2/SLC7A11/GPX4 pathway in ferroptosis regulation has been previously reported in DKD and other renal diseases [23, 32–34]. For example, quercetin and calycosin, key constituents of Abelmoschus manihot and Astragalus membranaceus respectively, have been shown to alleviate renal injury through regulation of NRF2-mediated ferroptosis signaling [23, 32, 33]. Moreover, Dodson et al. demonstrated that NRF2 plays a central role in limiting lipid peroxidation and ferroptosis under oxidative stress conditions [34]. These observations support a recognized role of NRF2 signaling in ferroptosis-associated renal injury. Compared with these earlier studies, the present work provides several additional insights. First, previous studies mainly focused on single bioactive compounds, whereas this study investigated a multi-component TCM formula. Second, this work integrated LC–MS/MS-based chemical profiling, network pharmacology prediction, and experimental validation in both in vivo and in vitro DKD models, providing a more comprehensive framework for exploring the mechanisms of complex herbal formulas. Third, by identifying 94 chemical constituents and linking them to ferroptosis-related pathways, this study provides a component-target-pathway perspective for understanding the pharmacological effects of ZXSZF. In addition, while many previous studies primarily focused on renal tubular injury, the present work specifically emphasizes podocyte ferroptosis, which represents a critical event in DKD progression.

To appropriately interpret these findings, the characteristics and limitations of the experimental model used in this study should also be considered. DKD was induced by unilateral nephrectomy combined with STZ administration, a commonly used experimental approach to reproduce hyperglycemia-associated renal injury. This model recapitulates several key pathological features of DKD, including proteinuria, glomerular hypertrophy, podocyte injury, and progressive renal dysfunction. However, it does not fully reproduce the chronic and multifactorial progression of human DKD, particularly the contribution of hypertension and long-term metabolic disturbances. Therefore, future studies should further validate the effects of ZXSZF in complementary DKD models, such as db/db mice and KK-Ay mice, as well as in models incorporating metabolic syndrome, hypertension, or genetic diabetic susceptibility, in order to better reflect the heterogeneous progression of human DKD. Such multi-model validation may help clarify whether the ferroptosis-regulating effect of ZXSZF remains consistent under distinct metabolic and hemodynamic backgrounds.

Network pharmacology was used in this study to explore potential targets and pathways associated with ZXSZF. Although LC–MS/MS analysis was first performed to identify chemical constituents and reduce reliance on database predictions, the predicted targets were not experimentally validated, which introduces the possibility of false-positive results. Furthermore, component-based network pharmacology focuses primarily on interactions between individual compounds and predicted targets and may not fully reflect the structural characteristics and synergistic interactions of complex TCM formulas. Although 94 compounds were identified by LC–MS/MS, only those with sufficient structural annotation and suitability for downstream database analysis were retained for network pharmacology. In addition, LC–MS/MS analysis may not detect all active constituents of ZXSZF, particularly compounds present at low abundance or macromolecular substances such as peptides and polysaccharides that are difficult to detect using this analytical platform [35]. These methodological limitations should therefore be considered when interpreting the network pharmacology results.

Future studies should focus on identifying the direct molecular targets of ZXSZF in DKD. Proteome-wide target identification technologies such as thermal proteome profiling (TPP) may provide valuable tools for this purpose [36, 37]. TPP enables high-throughput identification of drug-protein interactions in tissues or cell lysates without the need for chemical labeling, making it particularly suitable for studying complex herbal formulas [38, 39]. By applying TPP to renal tissues or podocyte lysates treated with ZXSZF, it may be possible to identify the direct and indirect binding targets of the formula. Integration of such target profiling data with bioinformatics resources such as IPA or Genevestigator may further facilitate target-effector association analysis and disease-related pathway mapping. These approaches may help clarify the authentic molecular targets and regulatory networks of ZXSZF, reduce uncertainties associated with purely computational predictions, and provide deeper insight into the molecular basis of its holistic therapeutic effects in DKD.

## Conclusion

The present study demonstrates that ZXSZF alleviates renal injury and podocyte damage in DKD. Using an integrated strategy involving LC–MS/MS chemical characterization, network pharmacology analysis, and experimental validation in both animal and cellular models, the findings indicate that the protective effects of ZXSZF are linked to modulation of ferroptosis-related processes associated with the NRF2/GPX4 signaling pathway.

## Supplementary Information

Additional file1 (PDF 2829 KB)

Additional file2 (DOCX 19 KB)

## Data Availability

The datasets used and/or analysed during the current study are available from the corresponding author on reasonable request.

## Abbreviations

AGEs: Advanced glycation end products
ACSL4: Acyl-CoA synthetase long-chain family member 4
BUN: Blood urea nitrogen
DKD: Diabetic kidney disease
Fer-1: Ferrostatin-1
GO: Gene Ontology
GPX4: Glutathione peroxidase 4
GSH: Glutathione
HE: Hematoxylin and eosin
LC-MS/MS: Liquid chromatography-tandem mass spectrometry
MDA: Malondialdehyde
MPC5: Mouse podocyte cell
NRF2: Nuclear factor-erythroid 2-related factor 2
PAS: Periodic acid-Schiff
PPI: Protein–protein interaction
ROS: Reactive oxygen species
SLC7A11: Solute carrier family 7, member 11
STZ: Streptozotocin
TCM: Traditional Chinese medicine
TPP: Thermal proteome profiling
UACR: Urinary albumin-to-creatinine ratio
24h-UTP: 24-hour urinary total protein
VAL ZXSZF: Valsartan ZhiXiaoSanZheng formula
