# Supplementary material for: ZhiXiaoSanZheng formula ameliorates podocyte injury in diabetic kidney disease by inhibiting ferroptosis: integrated network pharmacology and experimental validation
Source: Chin Med. 2026 Apr 7;21:113. doi: 10.1186/s13020-026-01389-x (PMC13054979; doi:10.1186/s13020-026-01389-x)
Supplement: Supplementary file 1 — Additional file1 (PDF 2829 KB) [file 13020_2026_1389_MOESM1_ESM.pdf]

Figure 4A-Nephrin

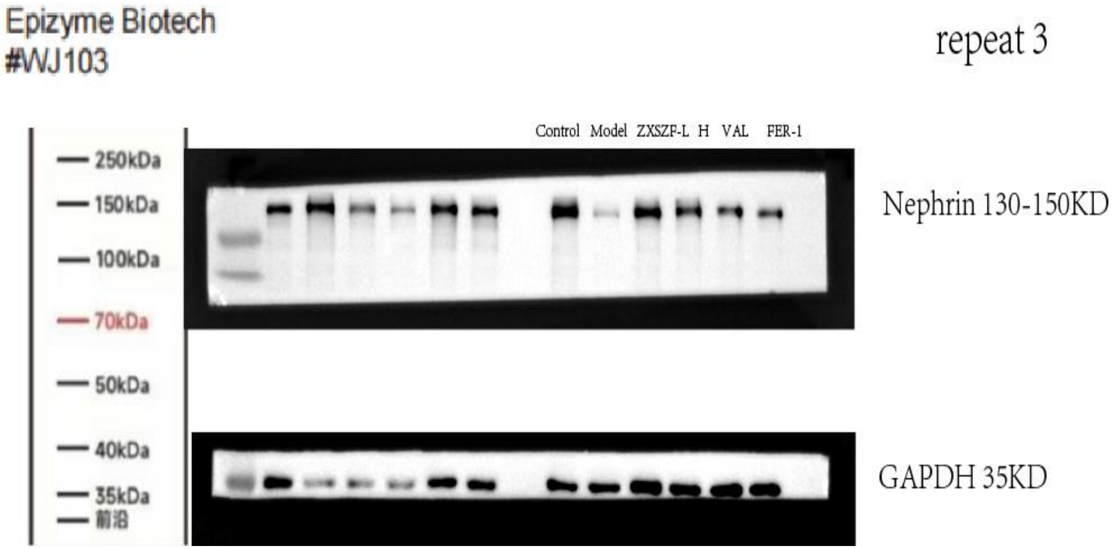

Original image (from the article)

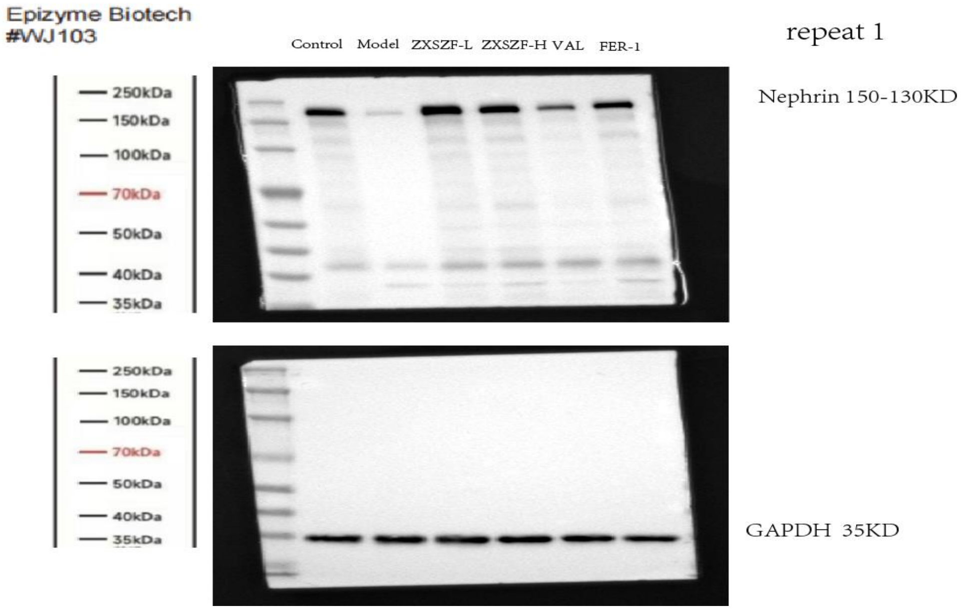

Repeated experimental image

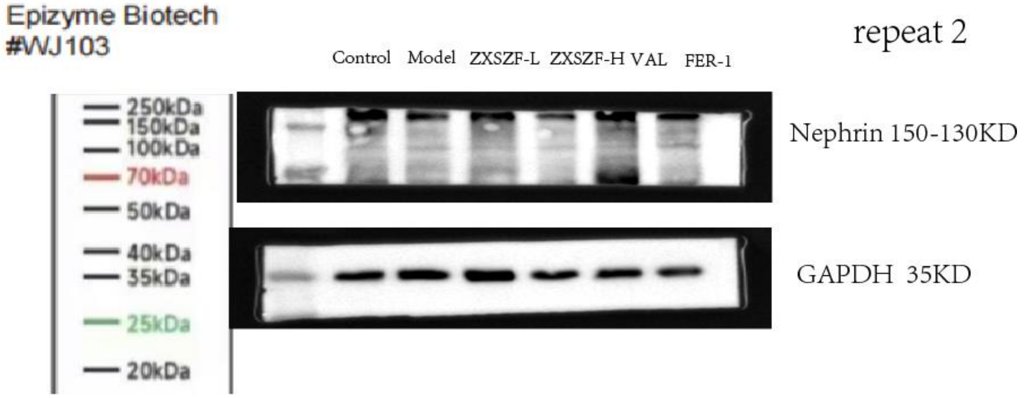

Repeated experimental image

Figure 4A-Desmin

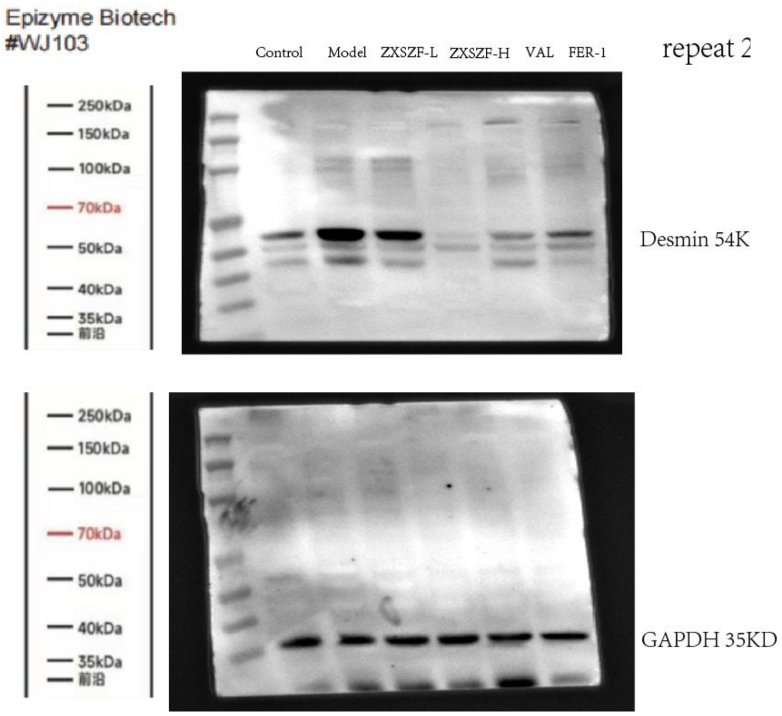

Original image (from the article)

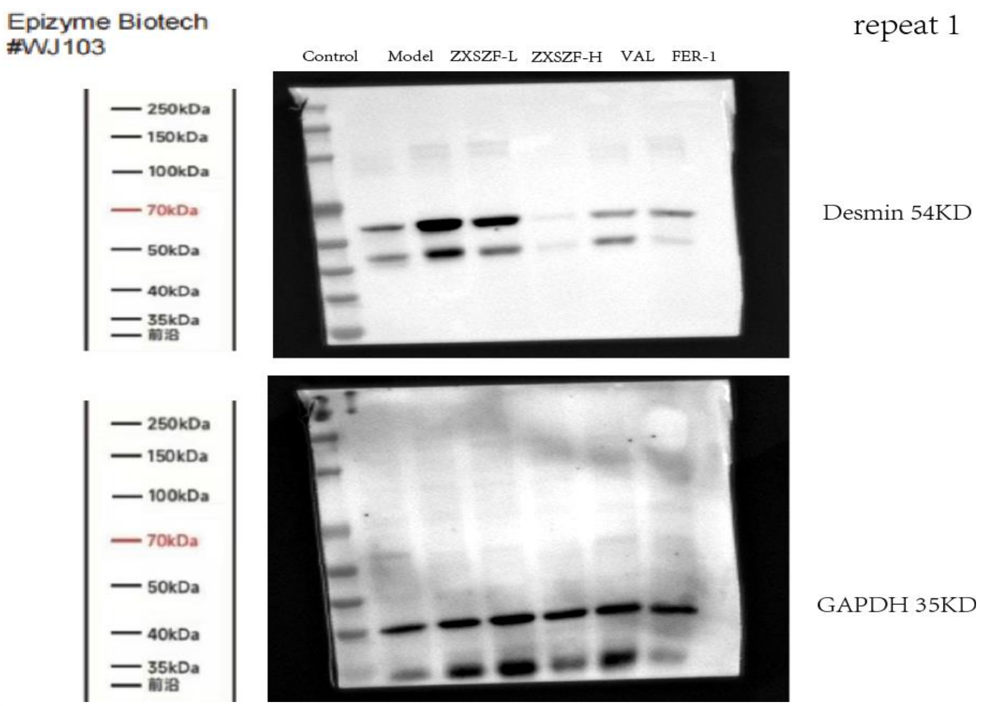

Repeated experimental image

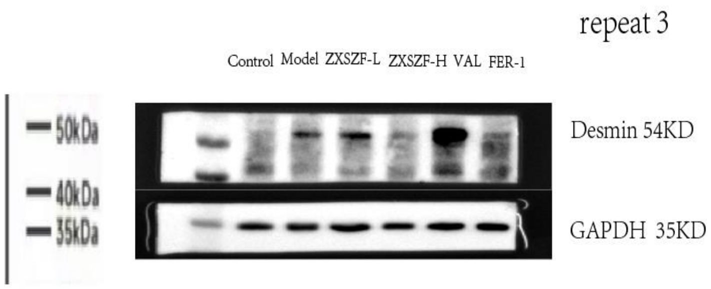

Repeated experimental image

Figure 4G-SLC7A11

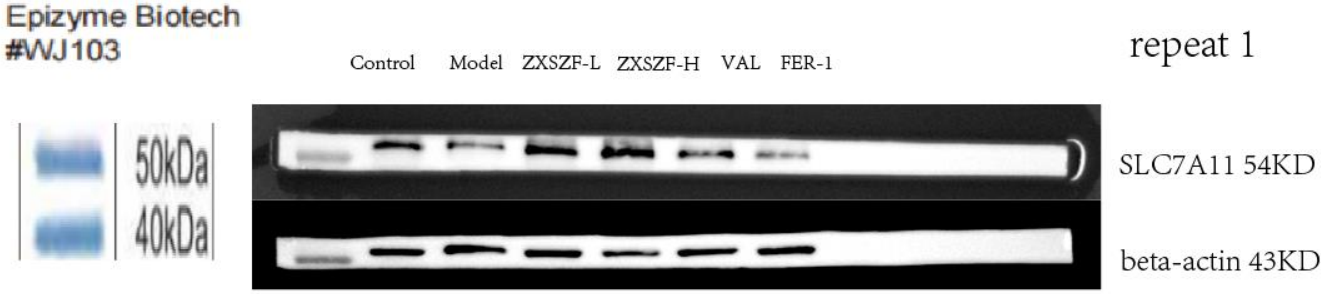

Original image (from the article)

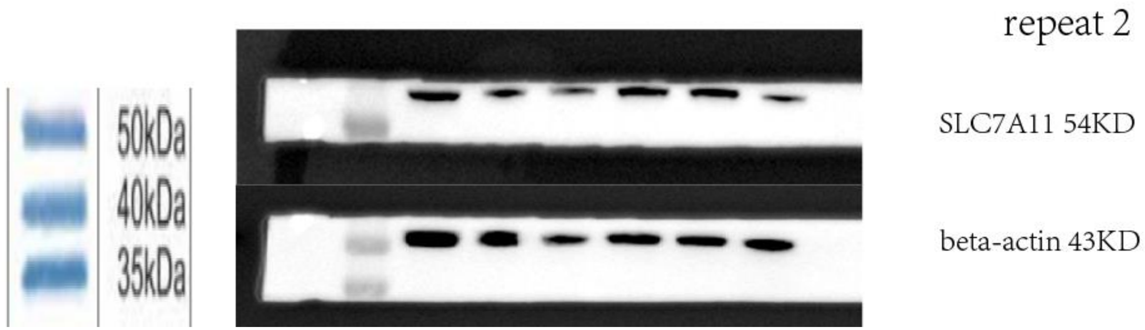

Repeated experimental image

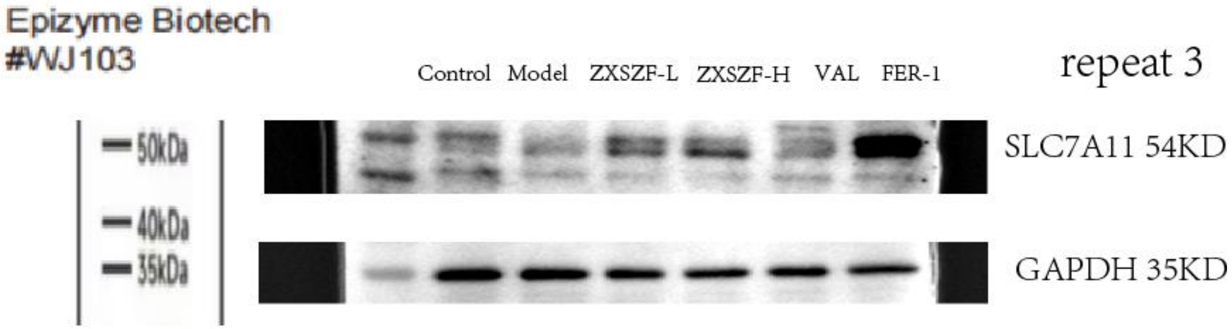

Repeated experimental image

Figure 4G-ACSL4

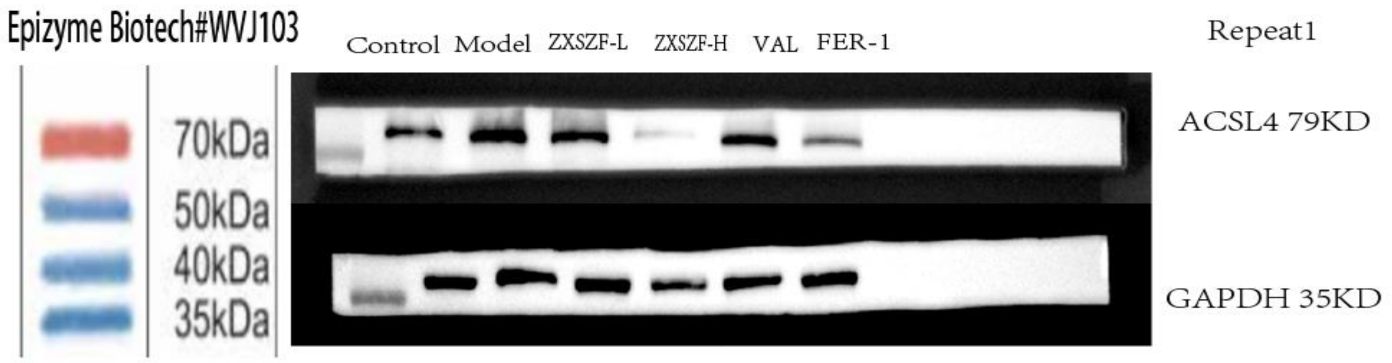

Repeated experimental image

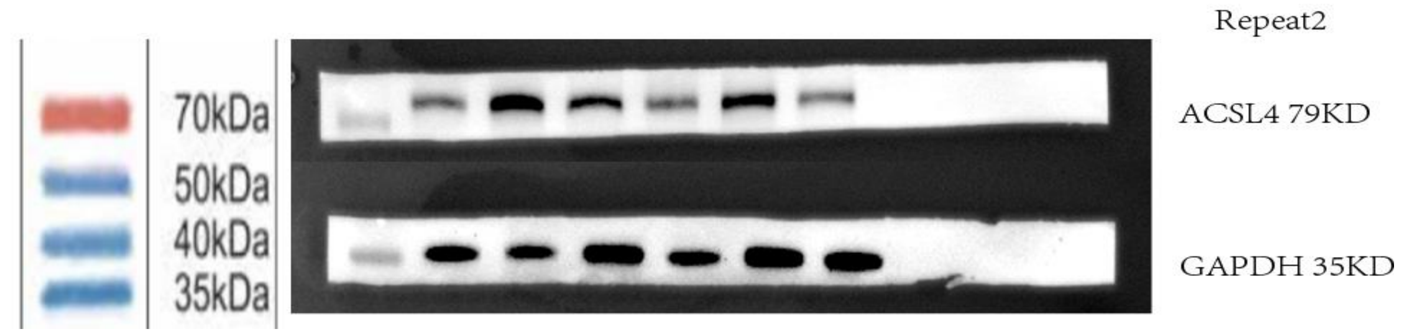

Original image (from the article)

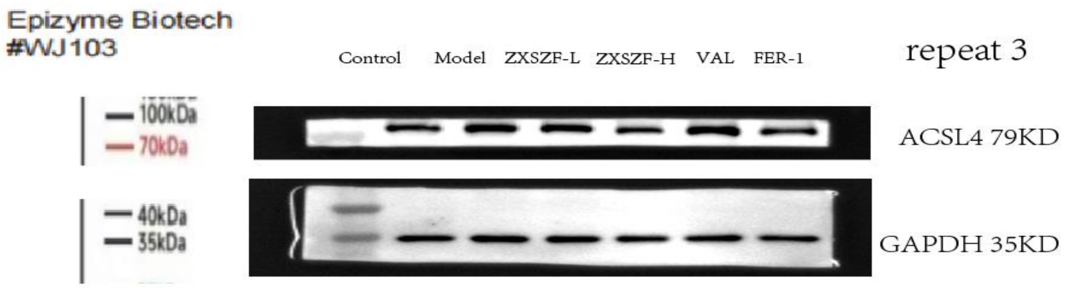

Repeated experimental image

Figure 4G-GPX4

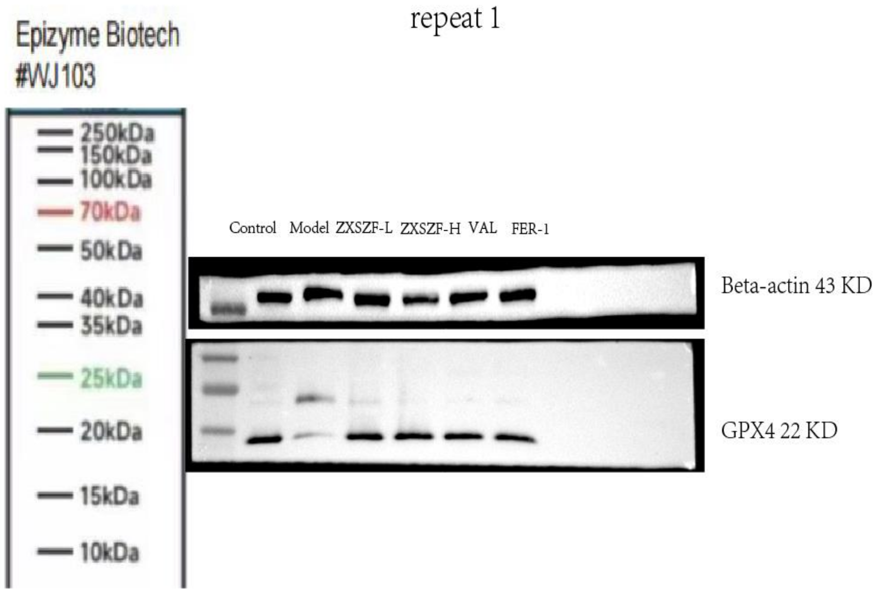

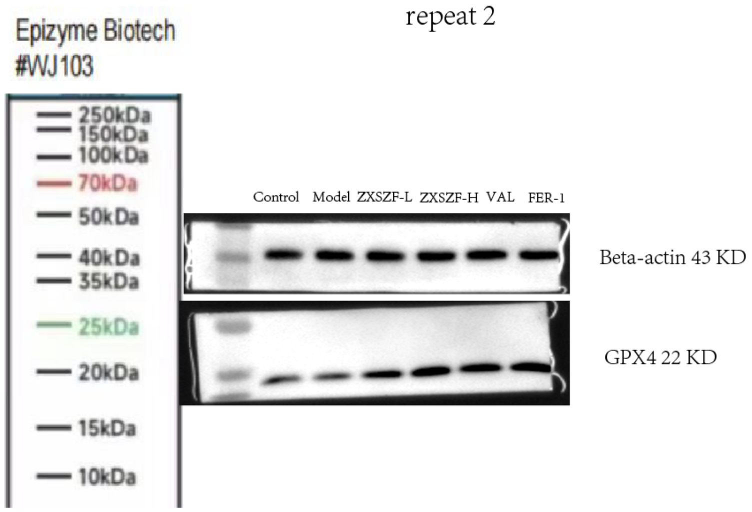

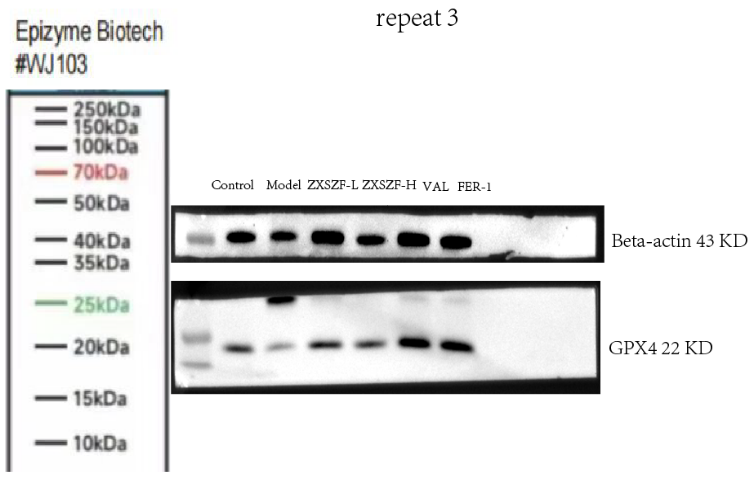

Original image (from the article)

Repeated experimental image

Repeated experimental image

Figure 4G-NRF2

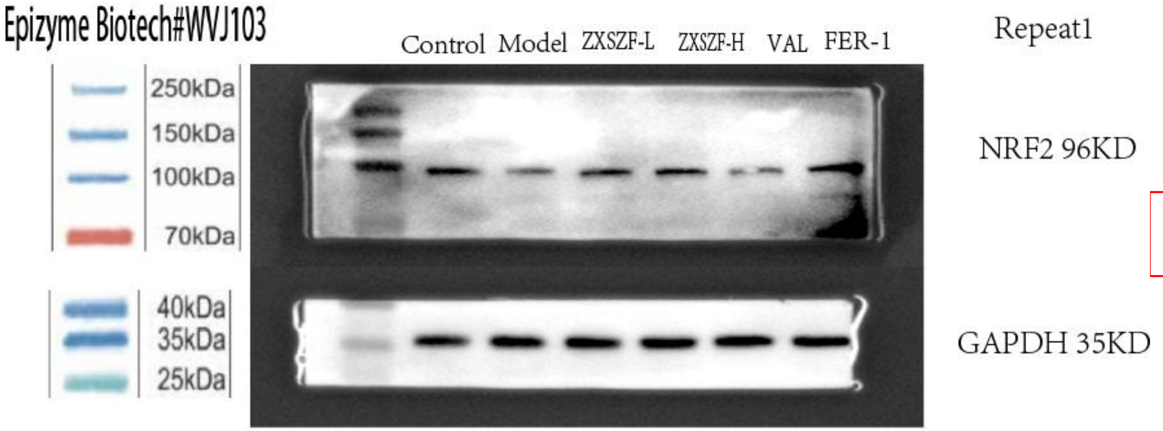

Original image (from the article)

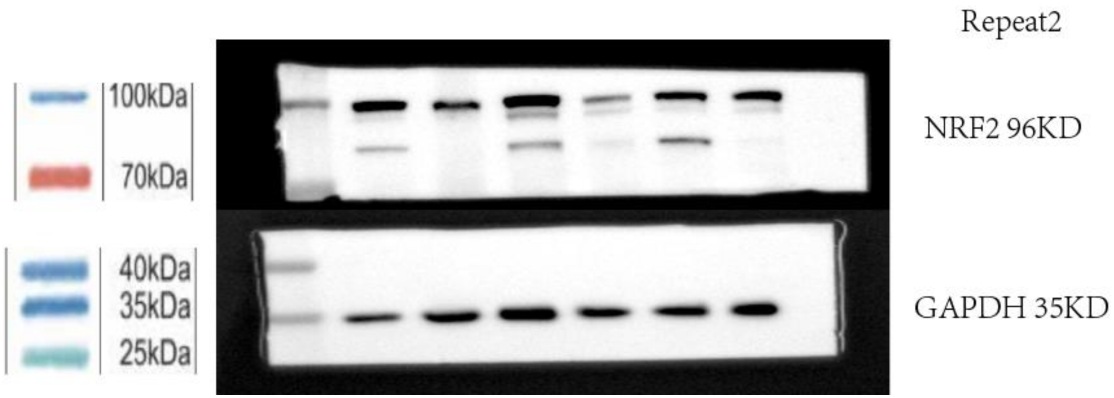

Repeated experimental image

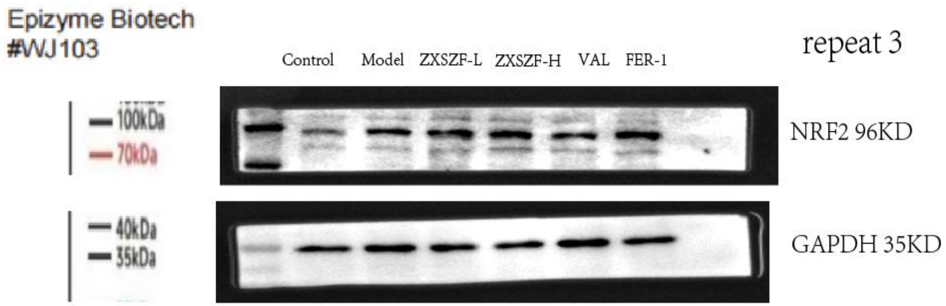

Repeated experimental image

Figure 5E-Nephrin

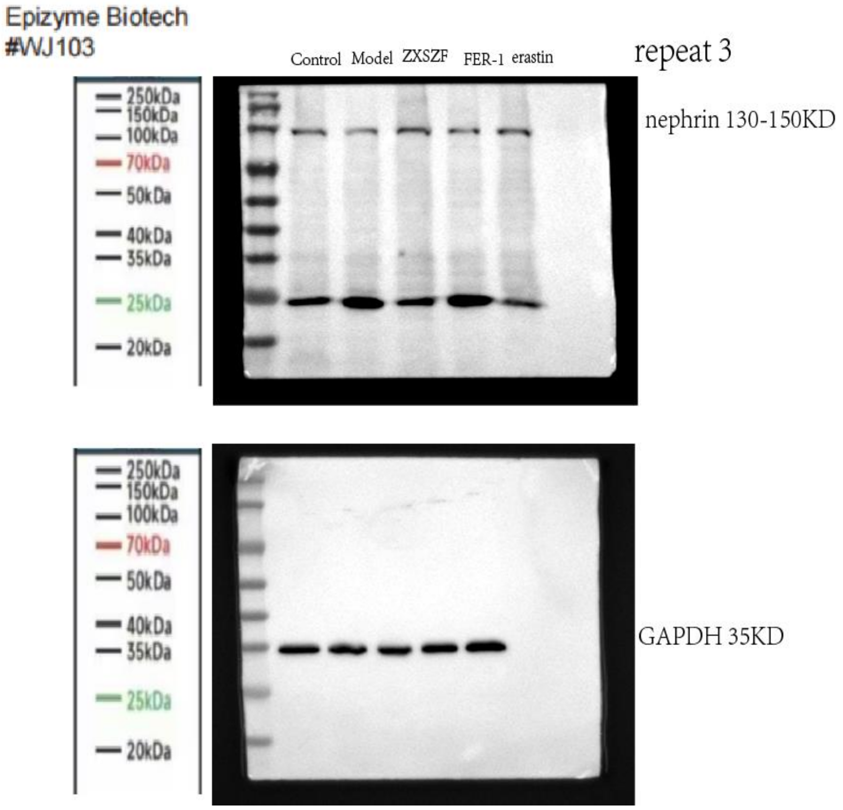

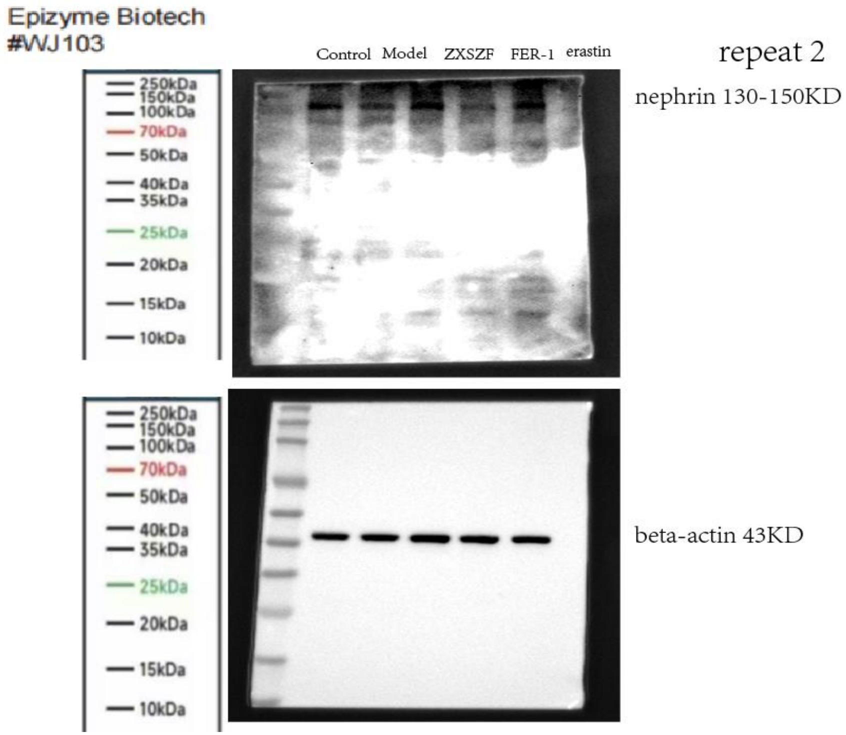

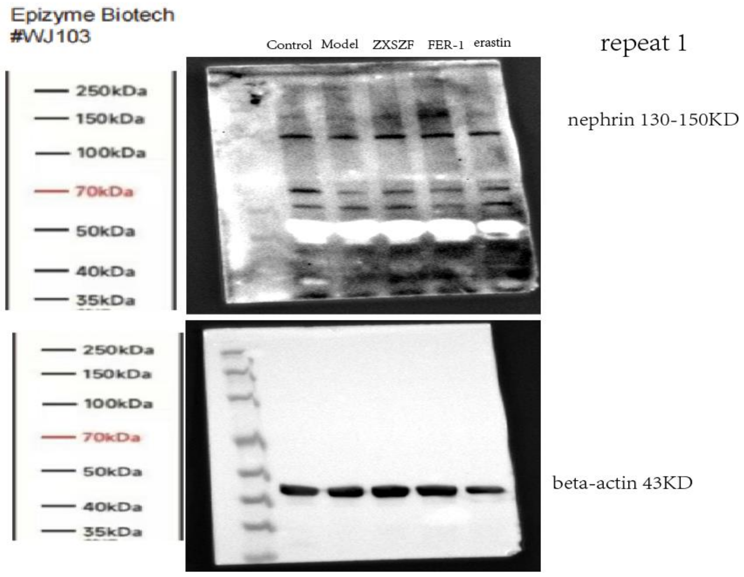

Original image (from the article)

Repeated experimental image

Repeated experimental image

Figure 5E-Podocin

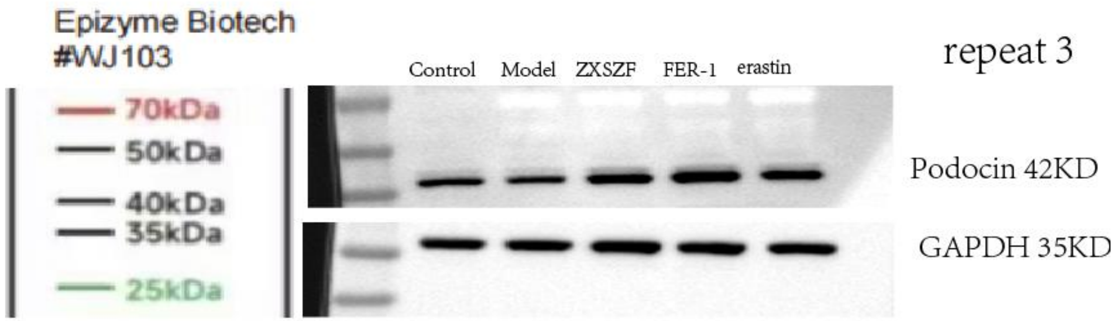

Original image (from the article)

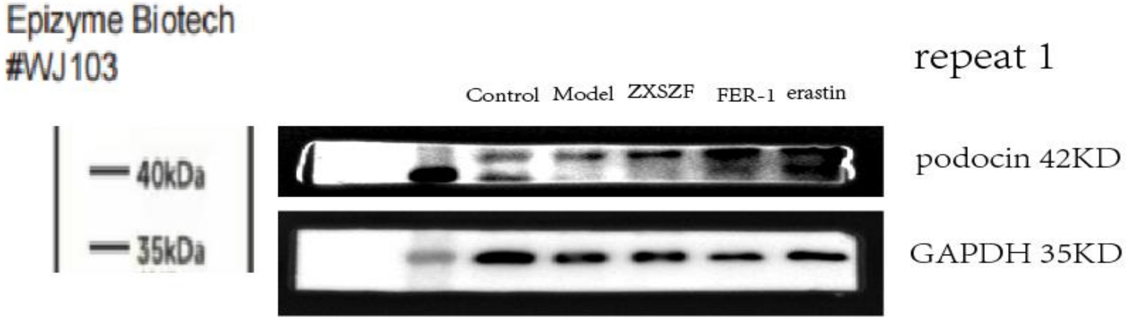

Repeated experimental image

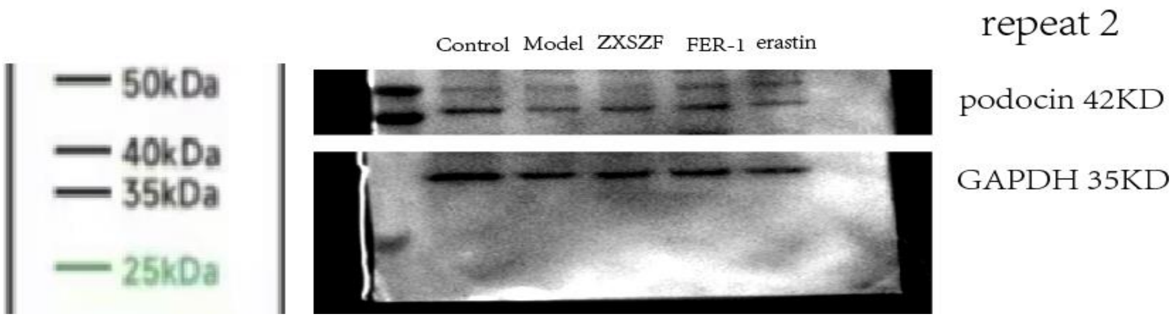

Repeated experimental image

Figure 6E-GPX4

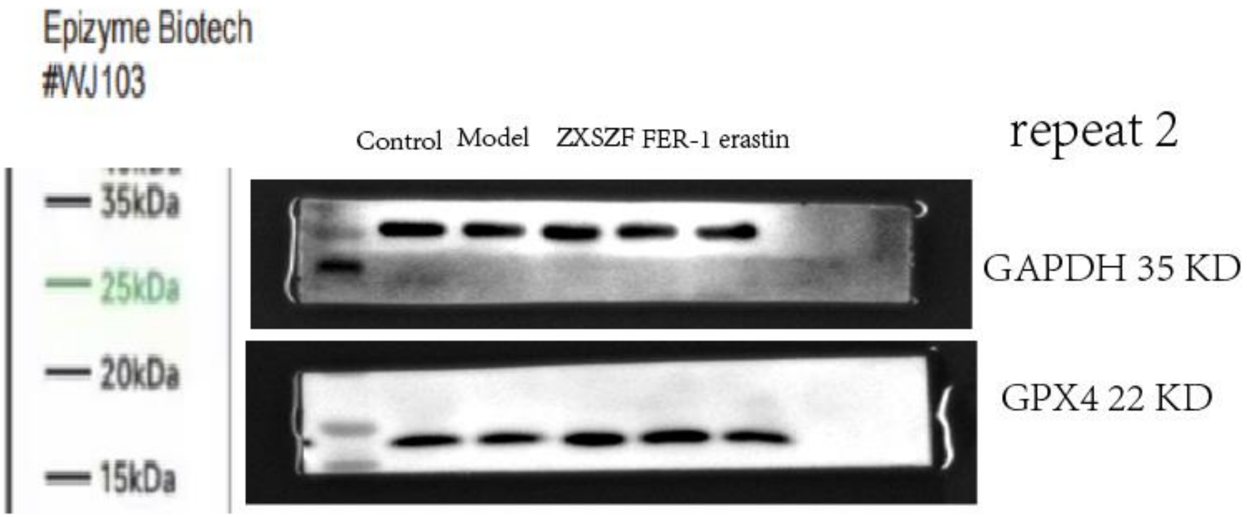

Repeated experimental image

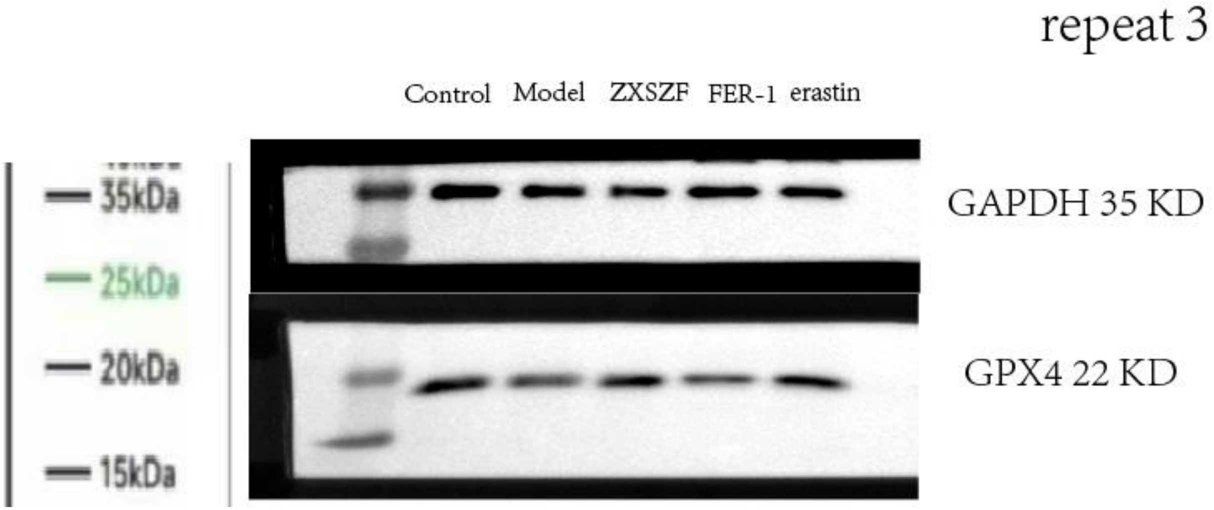

Original image (from the article)

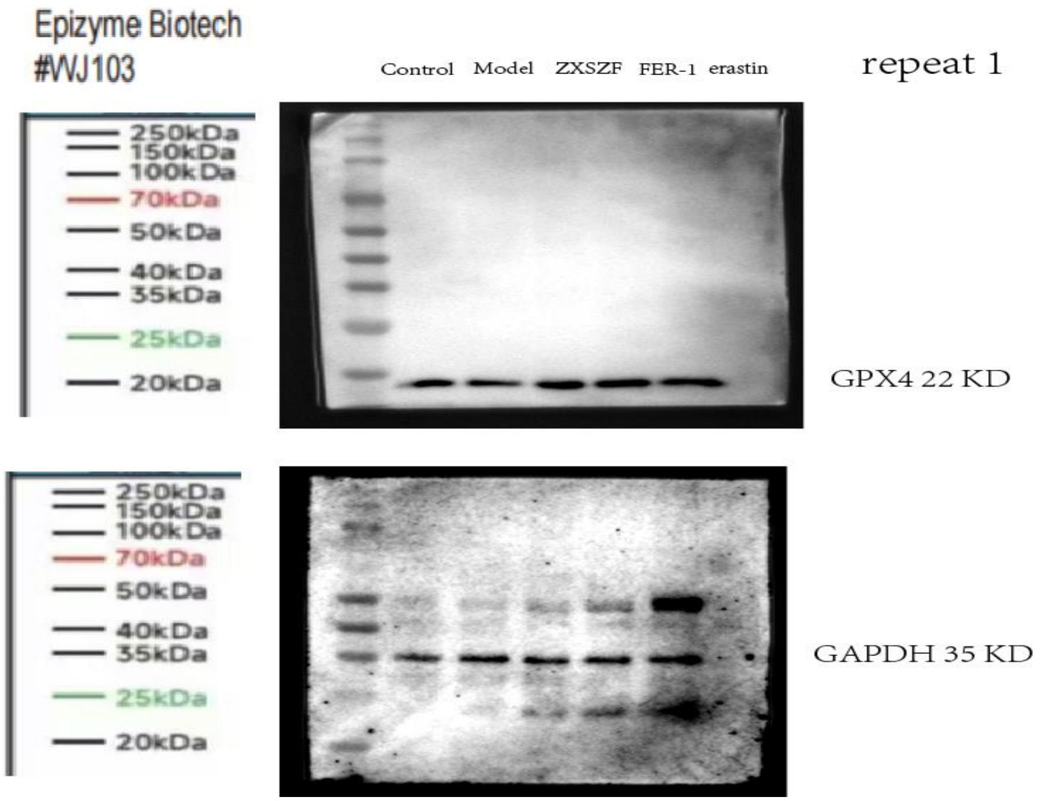

Repeated experimental image

Figure 6E-SLC7A11

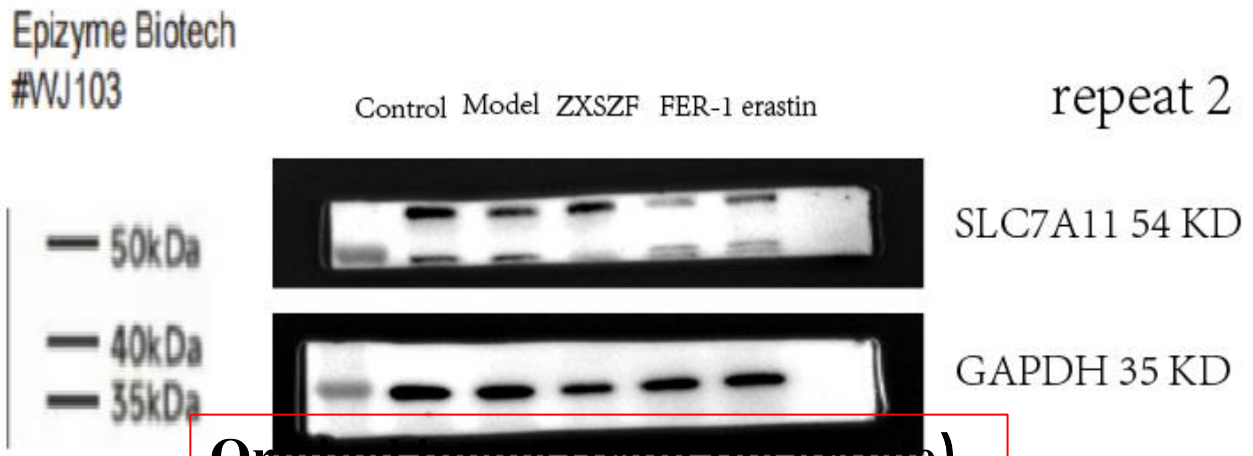

Original image (from the article)

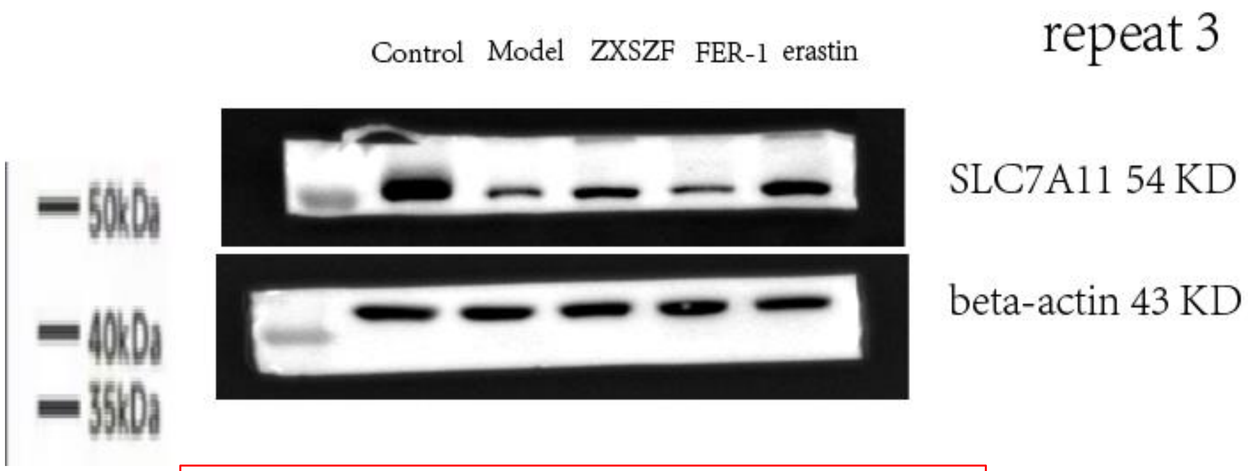

Repeated experimental image

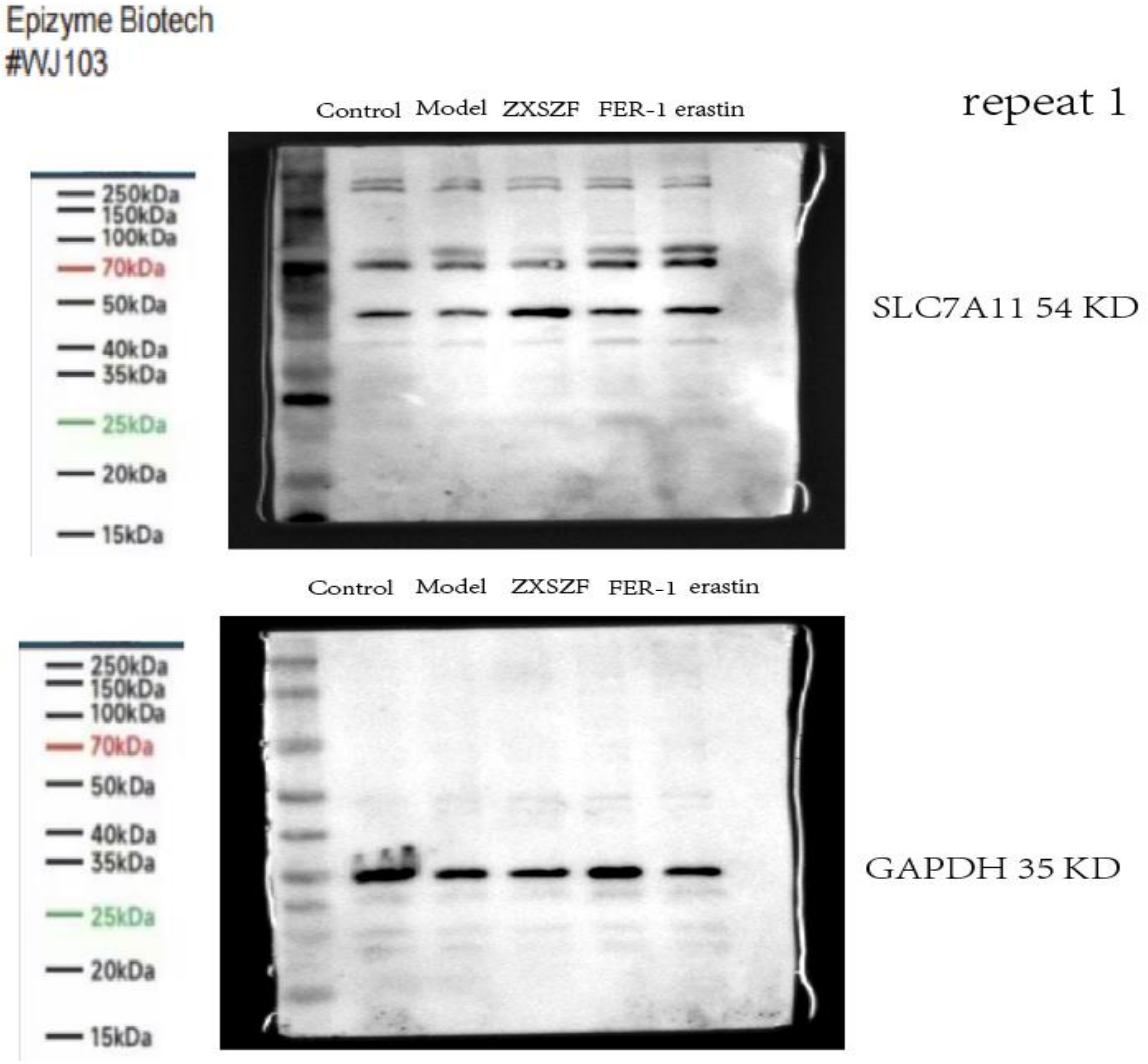

Repeated experimental image

Figure 6E-NRF2

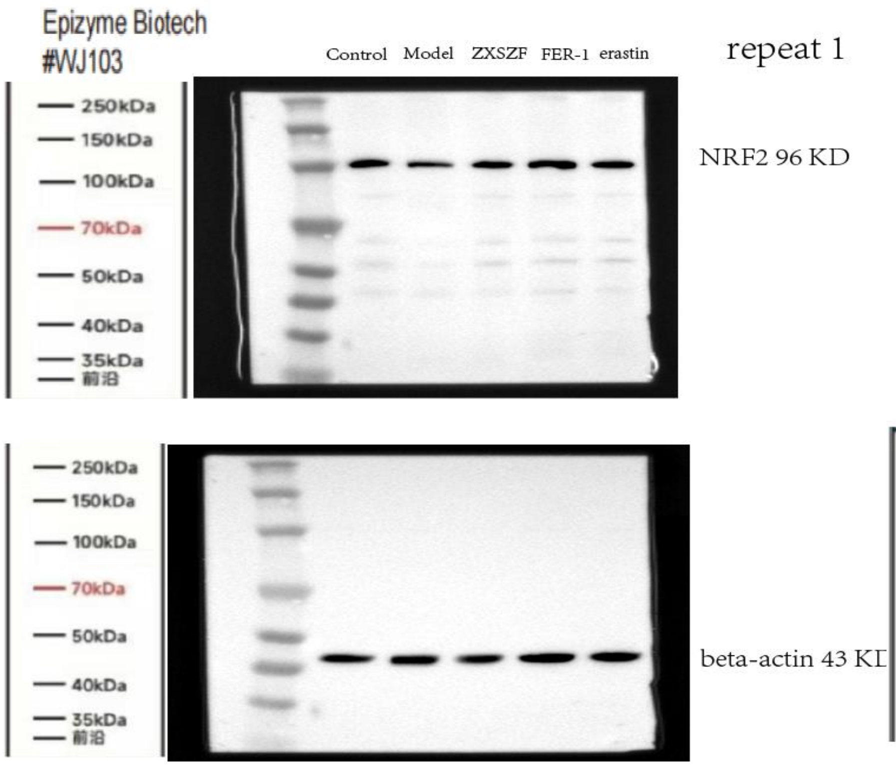

Original image (from the article)

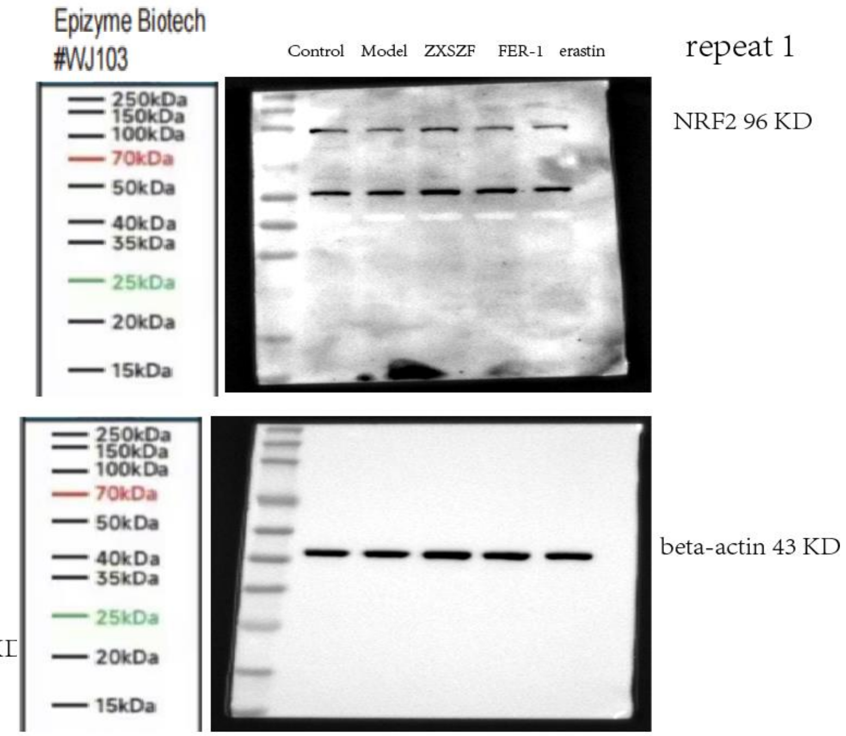

Repeated experimental image

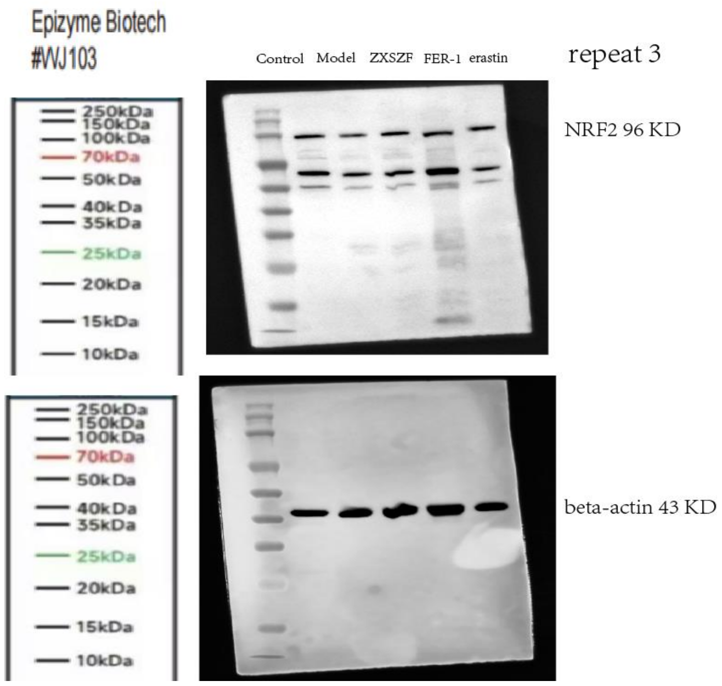

Repeated experimental image

Figure 6J-NRF2

Fig.6 F NRF2

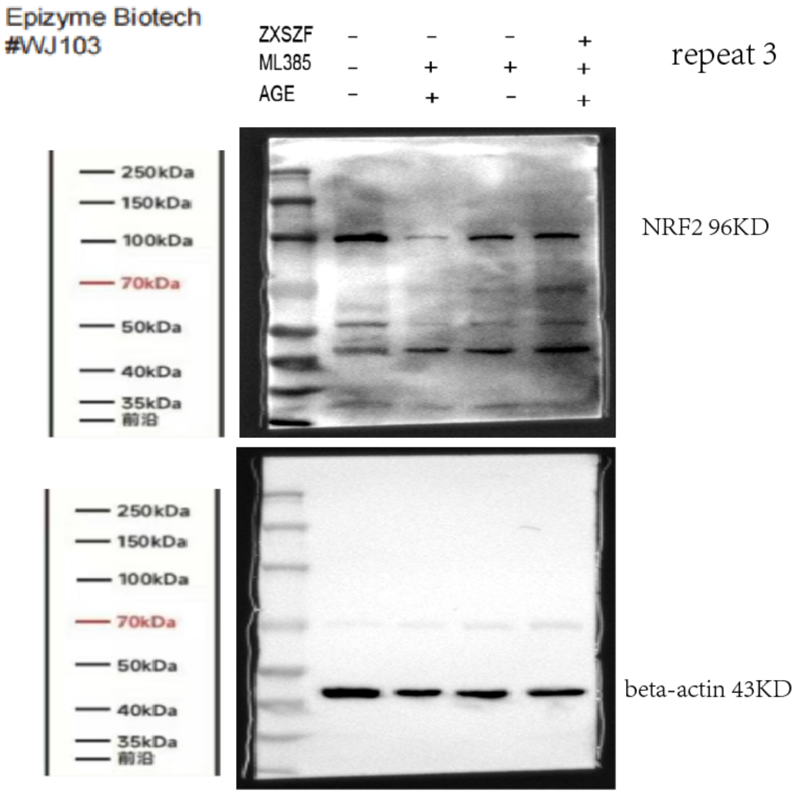

Original image (from the article)

Fig.6 F NRF2

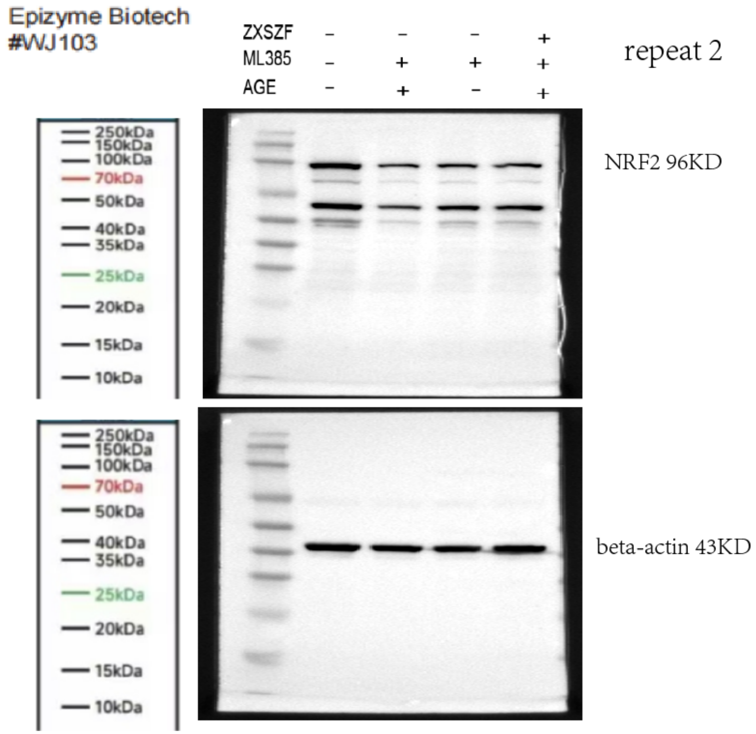

Repeated experimental image

Fig.6 F NRF2

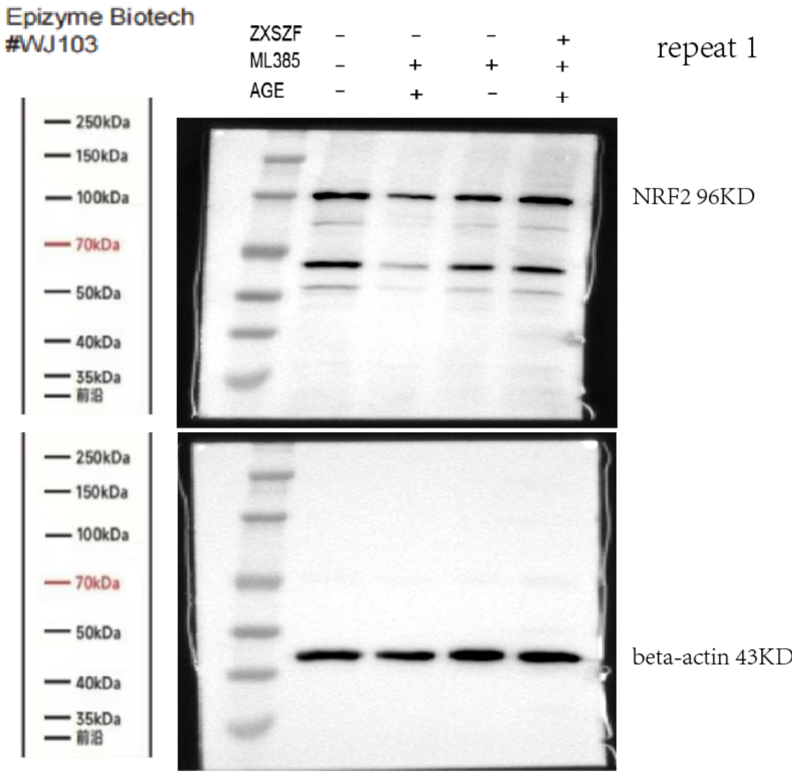

Repeated experimental image

Figure 6J-SLC7A11

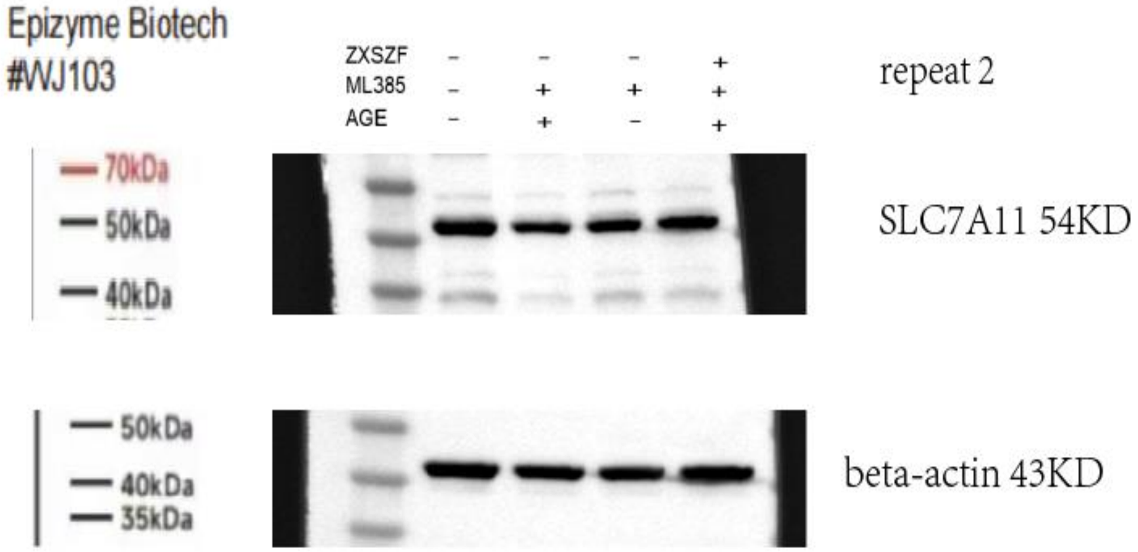

Original image (from the article)

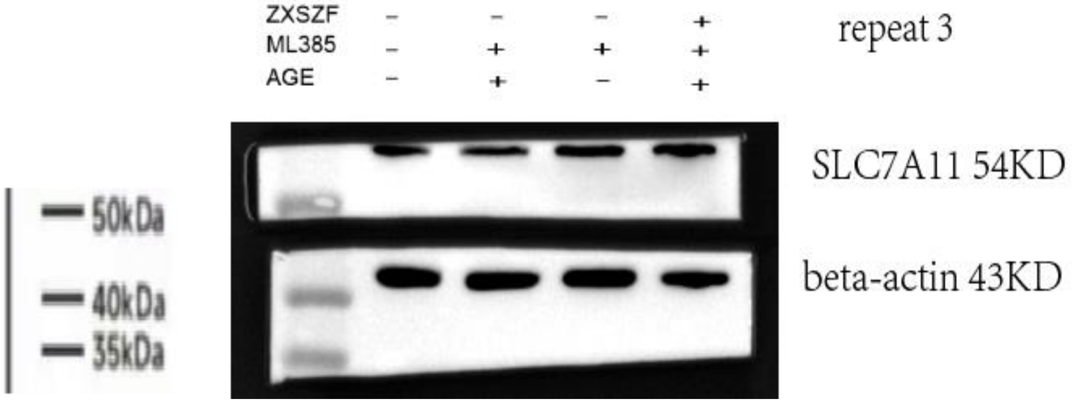

Repeated experimental image

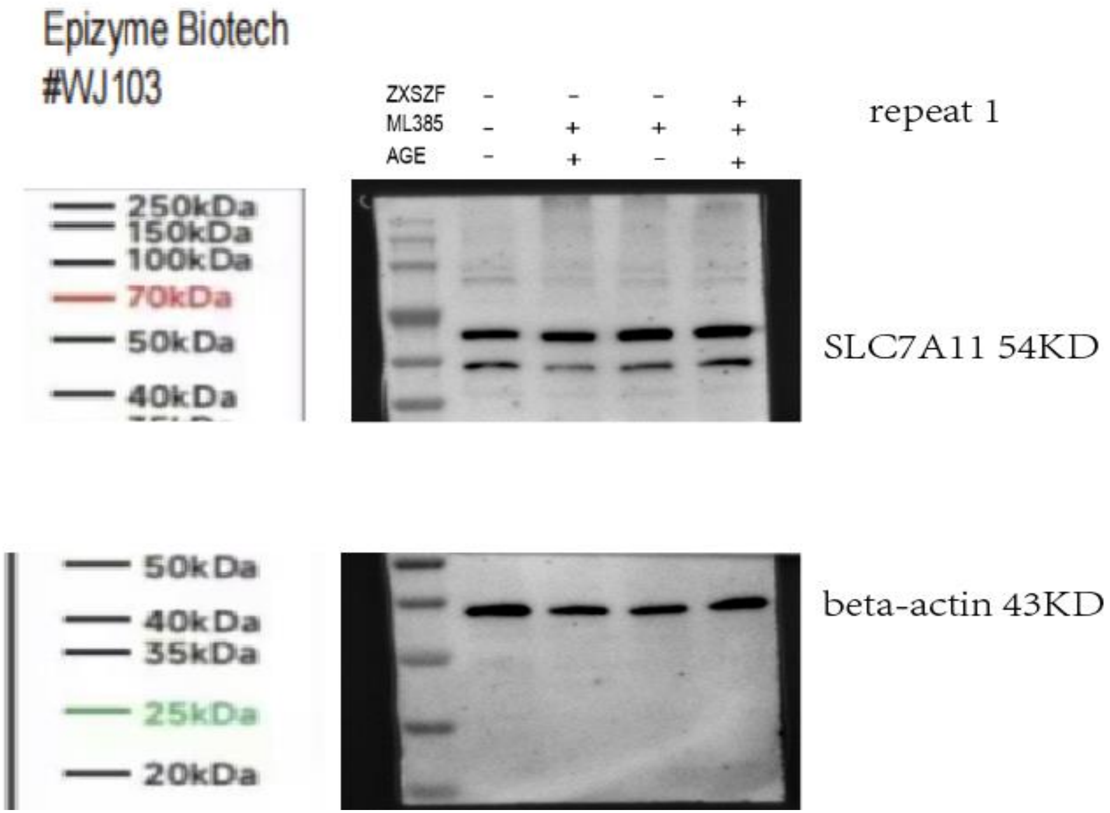

Repeated experimental image

Figure 6J-GPX4

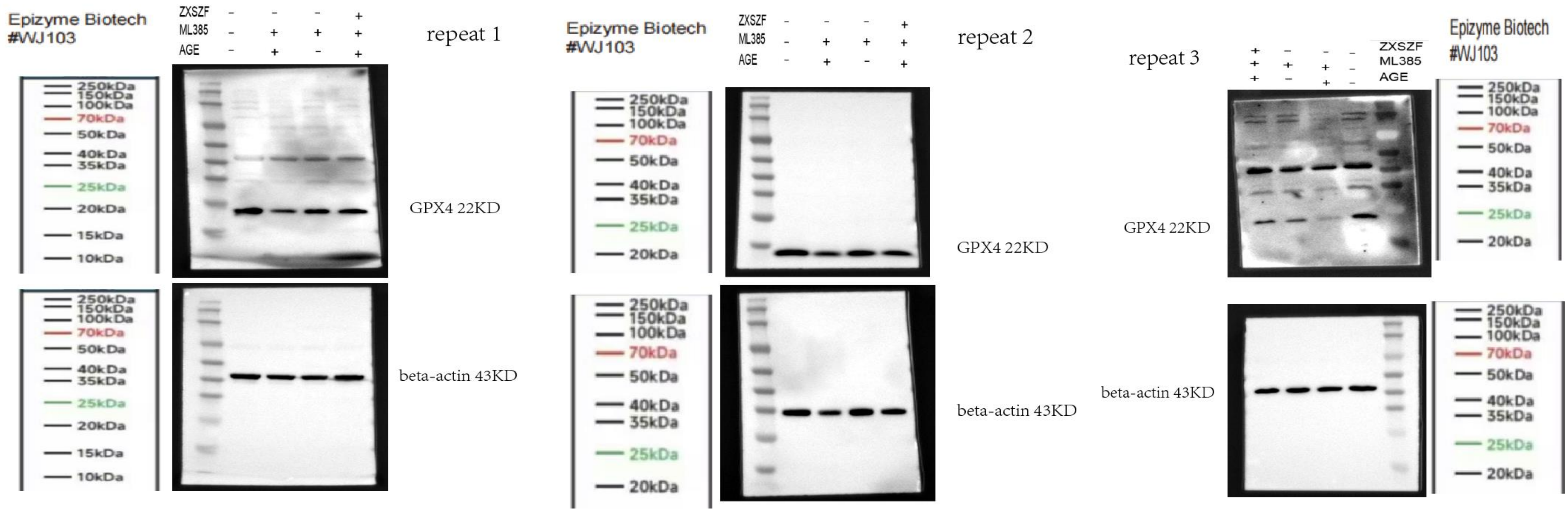

Original image (from the article)

Repeated experimental image

Repeated experimental image
