# Supplementary material for: ZhiXiaoSanZheng formula ameliorates podocyte injury in diabetic kidney disease by inhibiting ferroptosis: integrated network pharmacology and experimental validation
Source: Chin Med. 2026 Apr 7;21:113. doi: 10.1186/s13020-026-01389-x (PMC13054979; doi:10.1186/s13020-026-01389-x)
Supplement: Supplementary file 2 — Additional file2 (DOCX 19 KB) [file 13020_2026_1389_MOESM2_ESM.docx]

**Supplementary Method 1. Composition analysis of ZXSZF**

The samples were boiled and centrifuged, and 300 µL of supernatant was mixed with extraction solution and sonicated. After cooling, the samples were centrifuged, filtered, and stored at -80℃ for UHPLC-MS analysis. LC-MS/MS was conducted on a UHPLC system (U3000, Thermo Fisher Scientific) using a Waters ACQUITY UPLC HSS T3 column (1.8 μm, 2.1×100 mm). The sample injection volume was 10 μL, with a 0.3 mL/min flow rate. The mobile phases were 0.1% formic acid in water (A) and 0.1% formic acid in acetonitrile (B), and the gradient program was set as follows: 0 min, 100% A and 0% B; 10 min, 70% A and 30% B; 25 min, 60% A and 40% B; 30 min, 50% A and 50% B; 40 min, 30% A and 70% B; 45–60 min, 0% A and 100% B; 60.5–70 min, 100% A and 0% B. A Q Exactive Plus Orbitrap mass spectrometer (Thermo Fisher) with Compound Discover software was used in Full MS-ddMS2 mode, with separate scanning in positive and negative ion modes. The mass range was m/z 100–1200. The top 5 most abundant ions per cycle were screened for MS/MS. Key parameters were set as follows: sheath gas flow rate 40 L/min, aux gas flow rate 15 L/min, capillary temperature 320℃, auxiliary gas heating temperature 350℃, full MS resolution 70000, MS/MS resolution 17500, collision energy 30/40/50 NCE, and ion source voltage 3.2 kV.

**Supplementary Table 1.**

| **Table S1.** **List of antibodies used in this study** | | | | |
| --- | --- | --- | --- | --- |
| **Antibodies** | **Product code** | **Dilution** | **Application** | **Origin** |
| Nephrin | Ab216341 | 1:1000 | WB | abcam |
| Podocin （NPHS2） | Ab181143 | 1:1000 | WB | abcam |
| Desmin | Ab32362 | 1:1000 | WB | abcam |
| SLC7A11 | Ab307601 | 1:1000/1:100 | WB/IF | abcam |
| GPX4 | Ab125066 | 1:1000/1:100 | WB/IF | abcam |
| Nrf2 | YT3189 | 1:1000/1:100 | WB/IF | ImmunoWay |
| ACSL4 | 22401-1-AP | 1:1000 | WB | Proteintech |
| β-actin | 66009-1-Ig | 1:5000 | WB | Proteintech |
| GAPDH | 60004-1-Ig | 1:5000 | WB | Proteintech |
| HRP-conjugated goat anti-mouse IgG(H+L) | SA00001-1 | 1:8000 | WB | Proteintech |
| HRP-conjugated goat anti-rabbit IgG(H+L) | SA00001-2 | 1:8000 | WB | Proteintech |
| Goat anti-rabbit IgG Alexa Fluor 594 | A-32740 | 1:1000 | IF | Thermo Fisher |
| Goat anti-mouse IgG Alexa Fluor 594 | A-32742 | 1:1000 | IF | Thermo Fisher |
